# Successes and challenges in modeling heterogeneous BRAF^V600E^ mutated central nervous system neoplasms

**DOI:** 10.3389/fonc.2023.1223199

**Published:** 2023-10-18

**Authors:** Yao Lulu Xing, Dena Panovska, Claudia K. Petritsch

**Affiliations:** Department of Neurosurgery, Stanford University School of Medicine, Stanford, CA, United States

**Keywords:** BRAF V600E, brain tumor models, therapy resistance, melanoma metastases, low-grade glioma, tumor heterogeneity, tumor progression, tumor imaging

## Abstract

Central nervous system (CNS) neoplasms are difficult to treat due to their sensitive location. Over the past two decades, the availability of patient tumor materials facilitated large scale genomic and epigenomic profiling studies, which have resulted in detailed insights into the molecular underpinnings of CNS tumorigenesis. Based on results from these studies, CNS tumors have high molecular and cellular intra-tumoral and inter-tumoral heterogeneity. CNS cancer models have yet to reflect the broad diversity of CNS tumors and patients and the lack of such faithful cancer models represents a major bottleneck to urgently needed innovations in CNS cancer treatment. Pediatric cancer model development is lagging behind adult tumor model development, which is why we focus this review on CNS tumors mutated for BRAF^V600E^ which are more prevalent in the pediatric patient population. BRAF^V600E^-mutated CNS tumors exhibit high inter-tumoral heterogeneity, encompassing clinically and histopathological diverse tumor types. Moreover, BRAF^V600E^ is the second most common alteration in pediatric low-grade CNS tumors, and low-grade tumors are notoriously difficult to recapitulate *in vitro* and *in vivo*. Although the mutation predominates in low-grade CNS tumors, when combined with other mutations, most commonly CDKN2A deletion, BRAF^V600E^-mutated CNS tumors are prone to develop high-grade features, and therefore BRAF^V600E^-mutated CNS are a paradigm for tumor progression. Here, we describe existing *in vitro* and *in vivo* models of BRAF^V600E^-mutated CNS tumors, including patient-derived cell lines, patient-derived xenografts, syngeneic models, and genetically engineered mouse models, along with their advantages and shortcomings. We discuss which research gaps each model might be best suited to answer, and identify those areas in model development that need to be strengthened further. We highlight areas of potential research focus that will lead to the heightened predictive capacity of preclinical studies, allow for appropriate validation, and ultimately improve the success of “bench to bedside” translational research.

## 1 Introduction

### 1.1 BRAF^V600E^ mutation in central nervous system tumors have diverse prognosis and are candidates for molecular targeted therapy

The MEK-ERK signaling pathway is activated by growth factor-stimulated RAS binding to BRAF, which, when dysregulated, can initiate tumorigenesis by promoting uncontrolled cell growth (1) and cell fate changes (2, 3). Alterations in the BRAF gene, which encodes a central kinase in the MAPK signaling pathway, are prevalent in CNS tumors and are enriched in the pediatric population. BRAF protein is the most frequently mutated serine/threonine kinase in human cancer, with BRAF^V600E^ and KIAA1549::BRAF fusion being significant drivers of cellular transformation in pediatric CNS tumors, with high occurrences in low-grade gliomas (LGGs, up to 35%), and low occurrences in high-grade gliomas (HGGs, ~5% for BRAF^V600E^) (4, 5). BRAF^V600E^ is a class I mutation, the most common class of mutations in glioma. Through a somatic point mutation at codon V600, valine is substituted by glutamate (BRAF^V600E^), which is thought to mimic regulatory phosphorylation in the activation segment of the protein kinase domain (6). Consequently, BRAF^V600E^-mutated kinase monomers constitutively activate the downstream MAPK-ERK signaling pathway, regardless of any external stimuli and independent of upstream Ras (7, 8).

Due to the high incidence of BRAF^V600E^ in several solid cancers, small molecule inhibitors against BRAF^V600E^ have been developed and have been actively tested in the clinic (9–11). These BRAF inhibitors rapidly suppress MAPK signaling but exhibit only transient effects, prompting their subsequent combination with MAPK inhibitors for treatment (12). BRAF^V600E^ and MEK inhibitor combination therapy (e.g., dabrafenib+trametinib) has been tested in the clinic against glioma and clinical response rates of 70% and 33% have been observed amongst adults with low- and high-grade glioma, respectively. Similar response rates in children have led to the recent approval of BRAF^V600E^ inhibitor dabrafenib and MEK inhibitor trametinib for pediatric patients one year of age and older with BRAF^V600E^-mutated low-grade gliomas (LGG) who require systemic therapy (13–15). Despite these advances, further research is needed to develop new therapies and improve patient outcomes; clinical data shows tumor rebound when treatment is discontinued, and high-grade tumors frequently fail to respond or develop therapy resistance (16). Significant challenges stand in the way of improving current treatments, including that BRAF^V600E^-mutated pediatric LGG (pLGG) are frequently found with secondary mutations, most commonly CDKN2A deletion, and at lower frequency with single nucleotide variations (SNVs) in NF1, FGFR1, KRAS, and H3FA (5). In addition, 24% of BRAF^V600E^-mutated adult glioblastoma had mutations in the *TERT* promoter (17).

Alteration type influences the clinical course and therapy responsiveness of tumors. BRAF^V600E^ altered pLGG lacking tumor suppressor CDKN2A are at high risk for transforming into high-grade tumors when compared with BRAF^V600E^ CDKN2A balanced and BRAF wildtype tumors (18–20). Co-occurring alterations provide novel therapeutic vulnerabilities that can be targeted such as for example CDK4/6 inhibitors that target the p16/RB axis (21). Whether these drugs work synergistically or cooperatively with MAPK pathway inhibitors is not fully explored and additional preclinical testing is needed. Such preclinical testing requires preclinical models for BRAF^V600E^ CNS tumors that capture the genetic alterations found in human tumors. Mechanisms for cooperation of BRAF^V600E^ with CDKN2A loss for malignant progression have been investigated in genetically engineered mouse models (22). Other combinations of other co-occurring alterations (e.g., FGFR, H3FA) and BRAF^V600E^ are not yet available in models.

In conclusion, BRAF^V600E^ is a common kinase activating alteration in human cancer and in CNS with high prevalence in pLGG, whereby patients with BRAF^V600E^ altered tumors are routinely, successfully treated with molecular targeted therapy (BRAF inhibition). A unique feature is that BRAF^V600E^-mutated hemispheric pLGG tend to progress from low to high grade tumors at which point they become difficult to manage and exhibit therapy resistance to BRAF inhibition (20, 23). Models that recapitulate BRAF^V600E^ pLGG and the progression from low- to high-grade are not available, which is why little is known about their biology and the underlying mechanisms of progression; this will be discussed in detail in the “Developing models for tumor progression from low to high grade is challenging” section in this review.

The clinicopathologic diversity of BRAF^V600E^-mutated CNS tumors due to diverse co-occurring alterations makes treatment decisions ambiguous and raises the need for preclinical testing of combination therapies of BRAF/MEK inhibitors and therapies targeting co-occurring alterations. Such studies require generation of additional models which represent all types of genetic alterations combined with BRAF^V600E^ in human tumors.

### 1.2 BRAF^V600E^ -mutated CNS tumors striking inter-tumoral heterogeneity affects clinical prognosis

BRAF^V600E^ CNS tumors are enriched in the pediatric population and occur most frequently in pLGG (17-35% of pLGG) and are found in a diverse range of histopathologic subtypes of glioma. Both pleomorphic xanthoastrocytoma/PXA (40~80%), ganglioglioma/GG (25~45%) and desmoplastic infantile gangioglioma/astrocytoma (DIG/DIA; 45%) are more likely to harbor the mutation than other pLGG, such as pilocytic astrocytoma/PA (5~16%); optic pathway glioma (OPG; ~2%), dysembryoplastic neuroepithelial tumors/DNT (5~10%), infantile LGG (iLGG; 18%), and diffuse pediatric-type LGGs (19, 24–26). BRAF^V600E^ occurs at lower frequency in pHGG (6-14%) a histopathologically and clinical diverse group of tumors (4). Importantly, BRAF^V600E^ expression when coinciding with CDKN2A deletion marks a clinicopathologic subgroup of pLGG at high-risk for progression (5, 18). Consistent with this high risk, BRAF^V600E^ is the most frequent recurrent mutation in secondary pHGGs, occurring in 39% of cases (27).

BRAF^V600E^-mutated pLGG occurred most frequently in hemispheric regions (56%) but were also common in the diencephalon (29%). Interestingly, BRAF^V600E^-mutated iLGG which present most frequently in the midline rarely progresses, and the mutation is absent in infantile HGG (iHGG), which is paradoxical to its occurrence in pHGG overall (5, 26). These data suggest that tumor location affects the clinical course of BRAF^V600E^-mutated gliomas. Further to this point, BRAF^V600E^-mutated PA are prevalent in diencephalic areas in particular in unresectable hypothalamic/optic pathway PA, in contrast to KIAA1549-BRAF fusion-positive PA, which are prevalent in the cerebellum (24). Amongst these hypothalamic/optic pathway PA, BRAF^V600E^ is more frequently associated with non-progression, and KIAA1549-BRAF fusion was more common in progressive tumors (28).

BRAF-mutated diencephalic LGGs rarely present malignant transformation, but because they are usually unresectable, novel targeted therapy is strongly warranted. Indeed, clinical reports show promising results with BRAF^V600E^ inhibitor therapy in optic pathway glioma (29). Spatial distribution patterns of the BRAF^V600E^ mutation in pediatric, infantile, and adult LGGs (25) and clinical trial results with molecular targeted therapies against BRAF-mutated tumors have been extensively reviewed elsewhere (8, 17, 30, 31). While BRAF-fusion events have been associated with a good prognosis, and SNVs (most prominently BRAF^V600E^) have been associated with poor prognosis in pediatric LGG when all sites and histologies were included (5), the situation is reversed in BRAF-mutated pediatric diencephalic LGG such as optic pathway/hypothalamic glioma.

In conclusion, BRAF^V600E^-altered CNS tumors occur in diverse neuroanatomical locations, predominantly in hemispheric areas and BRAF^V600E^-altered PA exhibit a strong association with extra-cerebellar location. The microenvironment of tumors in distinct locations may determine the prognostic value of the BRAF alteration while responses to BRAF inhibitors appear to be universal. Due to the small study size of diencephalic tumors, further clinical and preclinical analyses are needed to test the neuroanatomical aspects of BRAF^V600E^-altered CNS tumors.

BRAF^V600^-mutated tumors present at a wide age range with a median age of 10.6 years in the pediatric population and are also found in adults. Genomic and epigenomic large scale analyses have detected BRAF^V600E^ in several adult CNS astrocytic tumors, including astroblastoma (24%-38%) (8, 25, 32), diffuse astrocytoma (DA; 30~40%), glioblastoma multiforme (GBM; ~ 6%) and astrocytoma (2~5%) (5, 17, 24, 33). Notably, the spectrum of histologic grades, pathology and nature of BRAF mutations in adults contrasted with that in pediatric tumors. In adults with BRAF-mutated CNS tumors, BRAF^V600E^ is most frequent in glioblastoma (~50%), and is common in epitheloid GBM (34, 35), followed by LGG (~22%), and PXA (18%). The mutation is notably absent in tumors with oligodendroglioma histology. Therefore, the histologic spectrum of BRAF^V600E^ CNS tumors is different in pediatric and adult patients. BRAF^V600E^ co-occurs with a similar set of alterations in pediatric and adult tumors, and both patient groups can benefit from BRAF-targeted therapy (17).

In non-CNS solid cancers, BRAF^V600E^ is frequently detected in malignant melanoma, hairy cell leukemia, colorectal carcinoma, and papillary thyroid carcinoma, as well as ovarian and lung tumors (36). Approximately 50% of melanomas harbor a BRAF^V600^ mutation (37, 38). Melanoma has the highest propensity to form brain metastases of all malignancies (39) and given that melanoma patients have a high incidence (10-40%) of developing metastases in the brain (40), the clinical relevance of BRAF mutational status in melanoma brain metastasis has been studied (41, 42). Evidence showed that patients with BRAF^V600E^-mutated melanoma were more likely to have CNS involvement than those with BRAF wild-type melanoma (43, 44). Clinical studies showed safety and efficacy of BRAF inhibitor therapy in patients with treated and untreated melanoma brain metastases more recently (45–47). In addition, BRAF and MEK inhibitor combination therapy provided promising clinical results against melanoma brain metastases (48, 49) which have prompted assessing baseline clinical features associated with outcomes (45, 46, 48–51). Genetic interaction studies in genetically engineered mouse models showed that BRAF ^V600E^ expression cooperates with Pten tumor suppressor loss to generate metastatic melanoma without CNS involvement (52). Expression of BRAF^V600E^ in Cdkn2a^Null^ mice generates melanomas without metastasis, but AKT1 activation promotes development of CNS metastases in this model (53).

In conclusion, BRAF^V600E^ expression is a driver mutation of CNS tumors that occur in various neuroanatomic locations and exhibit a wide range of age at presentation. In non-CNS cancers BRAF^V600E^ expression is not sufficient to generate CNS metastases and the type of co-occurring alterations determine whether CNS metastases form. More work is needed to unravel how BRAF^V600E^ mutation cooperates with co-alterations to increase invasive properties of tumor cells and generate brain metastasis.

### 1.3 Variation of BRAF^V600E^-mutated CNS neoplasms necessitates targeted and tailored strategies

Dysregulated neurodevelopmental programs underlying the distinct susceptibility of stem and progenitor cells to oncogenic alterations cause childhood CNS cancer (54). As stated above, the wide variance of disease location, age at onset, histopathology, and clinical outcome of BRAF^V600E^-mutated CNS neoplasms is quite striking and quite unique amongst pediatric CNS cancer types. This diversity sets BRAF^V600E^ apart from other point mutations in pediatric CNS cancers, such as the histone 3 variants H3.3 and H3.1, which are strongly associated with unique locations, high grade and more narrow age range, and a uniformly dismal prognosis (55). It suggests that cells at distinct stages and neuroanatomical locations can be susceptible to transformation by this oncogene (55, 56). How a single oncogene can generate such diverse tumors and lead to age-dependent differences of prognosis is unknown. Tumor extrinsic factors, such as the tumor microenvironment and the immune system may play an important role in the development of these tumors and these factors need to be considered as important determinants of tumor subtypes when developing BRAF^V600E^ CNS tumor models. We are far from capturing the diversity of BRAF^V600E^-mutated CNS tumor’s onset, histopathology, location, and prognosis with *in vitro* and *in vivo* models; thus, the underlying mechanisms leading to such diverse tumors remain poorly understood.

This represents a significant research problem. Despite the initial successes of treatment with FDA-approved first-generation small molecule inhibitors of BRAF^V600E^, such as vemurafenib, dabrafenib, and encorafenib, still therapeutic effects are not durable. Although effective in treating other varieties of BRAF^V600E^ malignancies, tolerance towards BRAFi as a monotherapy has been shown in melanoma and HGG, commonly through (acquired, secondary) reactivation of the MEK pathway in melanoma, and resistance mechanisms are discussed in more detail below. Since treatment efficacy is limited by drug resistance in HGGs and BRAF^V600E^ -mutated LGGs frequently progress to high-grade, novel therapeutic approaches against BRAF^V600E^ -mutated gliomas in general are needed, raising the urgent need for further refinement of BRAF^V600E^ models for preclinical studies and their successful translation.

Here, we aim at providing an overview of mammalian models for BRAF^V600E^ mutated CNS tumors; we list existing models, compare their usefulness for basic mechanistic studies and preclinical approaches, and describe current strategies to analyze and quantify tumor phenotypes and vulnerabilities. The objectives of this review are to categorize available preclinical *in vitro* and *in vivo* models of BRAF^V600E^-mutated CNS tumors. We highlight the applicability of these models for drug screening purposes and managing resistance. We describe their utility for disease characterization, including identifying and characterizing co-occurring mutations for their clinicopathologic characteristics and predictive and prognostic impact. In addition, we make suggestions on how to overcome existing limitations in model development. Preclinical studies should be performed in multiple models to avoid model-specific bias and ensure that preclinical data are robust and successfully translated to the clinic. Fortunately, several types of models are available and will be discussed in greater detail below.

## 2 *In Vitro* modeling and drug strategies

### 2.1 Cancer cell line models

Conventional cancer cell lines have been historically used for therapeutic screening due to their robust and uniform phenotypes and growth behavior as attached monolayer in high-serum culture conditions. Davies et al. identified BRAF^V600E^ mutations in established cancer cell lines DBTRG-05MG and AM38 (6), which subsequently became workhorses for preclinical testing, in particular of small molecule BRAF inhibitors, including PLX4720, a tool compound of Vemurafenib (**Table 1**). PLX4720 suppressed cell viability in several BRAF^V600E^ altered cell lines and patient-derived orthotopic xenografts (PDoX) from DBTRG-05MG and AM38, with PDoX recapitulating the *in vitro* sensitivity to BRAF inhibitors (**Table 2**) including PLX4720 monotherapy (79).

**Table 1:** A list of patient-derived cell lines representing various BRAF^V600E^-mutated tumor subtypes.

Cell Line Name	Cell Type	PDX	Sex	Age	Species	Other Alterations reported	Compounds Tested	Citations origin	Media conditions	Treatment reported	Marker reported	BRAF V600E mutation allelic status
2341	high-grade astrocytic	syngenic allograft (FVBN)			mouse	p16Ink/p14Arf homozygous deletion	dabrafenib + trametinib, PLX4720,	57	serum-free medium with growth factors	adenovirus- induced endogenous glioma	GFAP, Olig2, NF	heterozygous, downregulation of wildtype allele with passaging
STN-10049	Epitheloid GBM	yes	M	13Y	human	P53 and RB mutated	Dabrafenib, Trametinib	3				
SF10776	high-grade astrocytic	yes (mixed background)			mouse	p16Ink/p14Arf homozygous deletion	MK-1775 (wee1 inhibitor), XRT	58				
AM-38	GBM	yes	M	36Y	human	CDKN2A/B homozygous deletion, TERT (C250T), ALK (S737L)	PLX4720, PD0325901, selumetinib (refractory)	59	20% serum, attached	Local radiation, ACNU chemotherapy, rH-TNF	GFAP, S100	homozygous
AM38 R	GBM				human	isogenic to AM38	chronic exposure to vermurafenib in vitro	60				
DBTRG-05MG	GBM, recurrent	yes	F	59Y	human	PTEN homozygous deletion, CDKN2A/B homozygous deletion, chromosome 7 amp, TERT (C228T), POT1 (G40*)	PLX4720, PD-0332991 (cdk4/6i), BI2536, selumetinib (sensitive)	61	serum, attached, serum-free spheroids	Chemo- radiation,	vimentin, S100, neuron specific enolase, PDGFR, NCAM, GFAP	heterozygous
NMC-G1	Astrocytoma, Grade III	ND	F	NK	human	CDKN2A/B homozygous deletion, NF2 homozygous frameshift	PLX4720, trametinib	62	10% serum, DMEM			heterozygous
BT-40	juvenile Pilocytic Astrocytoma	yes	M	14Y	human		selumetinib (sensitive), XRT, trametinib	63				heterozygous
BT40TramR-A1 and -A3	juvenile Pilocytic Astrocytoma				human		trametinib	64	Stem cell medium with EGF/FGF, 3D			
BT76	juvenile Pilocytic Astrocytoma				human	isogenic to BT40	chronic exposure to vemurafenib	60				
NCH-MN-16	ND	yes	F	16Y	human		trametinib,	64				
IC-3635PXA	PXA, grade II	yes	F	10Y	human	CDKN2A deletion homozygous (trisomy chromosome 9 in PDX)	BRAF, BRAFV600Ei, MEKi, incl, PLX4720, dabrafenib (resistant), AZ628, vincristine, CHIR-265, SB590885	64	serum-free with EGFR/FGF		CD15, CD133, delining GFAP in cell line, vimentin	
MAF-794	AT/RT arising in a GG	no	M	6Y	human	INI-1 (SMARCB-1) loss	Autophagy inhibitor (bafilomycin) vemurafenib	60, 65	OptiMEM Reduced Serum media supplemented with 15% FBS and 1% penicillin/streptomycin			
MAF-794R	isogenic to MAF-794, resistant	no	M	6Y	human	isogenic to MAF794	Autophagy inhibitor (bafilomycin) vemurafenib,	60, 65	OptiMEM Reduced Serum media supplemented with 15% FBS and 1% penicillin/streptomycin			
MAF-905	ND	no	ND	ND	human		Autophagy inhibitor (bafilomycin) vemurafenib, LY3009120, belvarafenib, ZM336372	60	OptiMEM Reduced Serum media supplemented with 15% FBS and 1% penicillin / streptomycin			
PXA2/PXA654	PXA	ND	M	7.5Y	human		PVSRIPO immunotherapy	66			PVR CD155	
YMG62-P and R	High-grade glioma	yes	F	16Y	human	hTERT 228c>T promoter mutation, CDKN2A homozygus deletion, chromosome 7 copy number gains	HSP90 inhibitor	67	serum-free EF20	XRT, TMZ, Dabrafenibplus Trametinib (resistant)		
YMG89-P and R	High-grade glioma	yes	M	26Y	human	hTERT promoter mutation, CDKN2A homozygus deletion, chromosome 7 copy number gains	HSP90 inhibitor	67		Dabrafenib		
YMG33	GBM				human		HSP90 inhibitor	67				
YMG209	aPXA				human		HSP90 inhibitor	67				
NGT41	epitheloid GBM, disseminated to spinal cord	yes	M	57Y	human	hTERT (C228T) promoter mutation, CDKN2A heterozygous deletion, chrom 7 gains	dabrafenib (sensitive)	68	from CSF, OptiMEM = serum	radiation + TMZ, BRAF+MEKi,		
3T3 BRAFv600E	ND	no			human	TERT promoter (C250T), CDKN2A/B loss	PLX4720	69				ectopic retroviral expression
VBT92	aPXA+	no			human	TERT (C228T), CDKN2A loss	dabrafenib, YK-4-279	70	serum attached			pos
VBT125	GSo	no			human	TERT (C228T), CDKN2A loss	YK-4-279	70	serum attached			pos
BTL1304	GS	no			human	TERT (228T), PTEN (K266E)		70	serum attached			pos
BTL2231	PXA#	no			human	CDKN2A loss		70	serum attached			pos
VBT150	PXA	no			human	wt		70	serum attached			pos
VBT172	aPXA	no			human			70				pos

P: prior treatment; ND: not determined; NK: not known; R: relapse; M: male; F: female.

**Table 2:** Established resistant preclinical models of BRAF^V600E^ melanoma and colorectal cancer.

Model Type/Name		BRAF V600E		Tumor Type		other mutations		Drug details		Treatment strategy		Dose		Time to resistance		Clonal selection		Resistance driver		Citation		Pathway reactivation	
PDCL																							
WM989		Yes		Melanoma				Vemurafenib		Continuous		1 μM		1-4 weeks		Single-cell derived clones		increased expression AXL, EGFR, FGFR1, PDGFRB		71			
WM983B		Yes		Melanoma				Vemurafenib		Continuous		1 μM		1-4 weeks		Single-cell derived clones		increased expression AXL, EGFR, FGFR1, PDGFRB		71			
M229		Yes		Melanoma				PLX4032 (Vemurafenib)		Increasing continuous		0.01 - 10 µM		NA		None		mutually exclusive PDGFRB, N-RAS upregulation		72		MAPK	
M249		Yes		Melanoma				PLX4032 (Vemurafenib)		Increasing continuous		0.01 - 10 µM		NA		None		mutually exclusive PDGFRB, NRAS upregulation		72		MAPK	
M238		Yes		Melanoma				PLX4032 (Vemurafenib)		Increasing continuous		0.01 - 10 µM		NA		None		mutually exclusive PDGFRB, NRAS upregulation		72		MAPK	
M249 (VemR and CTLR)		Yes		Melanoma		MART-1 27-35; HLA A*0201		Vemurafenib and MART- specific CTL (F5 CTL)		Increasing continuous		0.1-10 μmol/L		3 months		None		Protein levels of EGFR, c-KIT, Met, and PDGFRβ were upregulated, and IGFRβ was downregulated		73		RTK	
M238 (VemR and CTLR)		Yes		Melanoma		/		Vemurafenib and MART- specific CTL (F5 CTL)		Increasing continuous		0.1-10 μmol/L		3 months		None		EGFR levels were significantly upregulated whereas PDGFRβ levels were reduced. Protein levels of Met and IGFRβ remained unchanged		73		RTK: during the course of acquisition of BRAFi resistance, melanomas develop cross- resistance to CTL- and NK-killing.	
M249 (VemR and CTLR)		Yes		Melanoma		MART-1 27-35; HLA A*0201		Vemurafenib and MART- specific CTL (F5 CTL)		Increasing continuous		F5 CTLs ( (E:T 20:1, 40:1, 60:1)		8 weeks		Two rounds of limiting dilution series and single-cell		Protein levels of EGFR, c-KIT, Met, and PDGFRβ were upregulated, and IGFRβ was downregulated		73		RTK: during the course of acquisition of BRAFi resistance, melanomas develop cross- resistance to CTL- and NK-killing.	
M238 (VemR and CTLR)		Yes		Melanoma		/		Vemurafenib and MART- specific CTL (F5 CTL)		Increasing continuous		F5 CTLs ( (E:T 20:1, 40:1, 60:1)		8 weeks		Two rounds of limiting dilution series and single-cell		EGFR levels were significantly upregulated whereas PDGFRβ levels were reduced. Protein levels of Met and IGFRβ remained unchanged		73		RTK: during the course of acquisition of BRAFi resistance, melanomas develop cross- resistance to CTL- and NK-killing.	
HMEX1906		Yes		Melanoma		BRAF T1179A		Vemurafenib		Continuous		50 nM		10 days		None		BRAF amplification		74		ERK signaling and decreased proliferation	
45V-RT *		Yes		Melanoma		/		Vemurafenib		Continuous		50 nM		10 days;		None		BRAF amplification; could only be grown in presence of 50nM vemurafenib		74		ERK signaling and decreased proliferation	
PDX																							
HMEX1906		Yes		Melanoma		BRAF T1179A		Vemurafenib		Continuous		45 mg/kg twice daily		8 weeks		None		BRAF amp		74		ERK signaling	
45V-RT		Yes		Melanoma				Vemurafenib		Continuous		45 mg/kg twice daily				HMEX1906 resistant subline reimplanted into mice				74		ERK signaling	
HMEX1906		Yes		Melanoma		BRAF T1179A		Vemurafenib		Continuous vs Intermittent		15 mg/kg twice daily (continuous); 4 weeks on/ 2 weeks off (intermittent)		100 days for continuous; no resistance after 200 days for intermittent		None				74		ERK signaling	
HMEX2613		Yes		Melanoma		/		Vemurafenib		Continuous vs Intermittent		45 mg/kg twice daily				None				74		ERK signaling	
Cell lines																							
WM239A		Overexpressed		Metastatic melanoma		/		Encorafenib		Continuous vs Intermittent		500 nM vs 7 days on + 7 days off (4 weeks in total)		2 weeks		None		increased Nf-kB, EMT, hypooxia		75			
Colo205		Yes		Colorectal		/		PLX4720		Continuous		5 μmol/L		Until 90% confluency		None		EGFR and KRAS amplification; AKT activation		76		(PI3K)/ Akt	
HT29		Yes		Colorectal		/		PLX4720		Increasing continuous		0.1, 0.5, 1, 2, and 4 μmol/L		Until 90% confluency		One resistant clone was grown for 2 months		EGFR and KRAS amplification; AKT activation		76		(PI3K)/ Akt	
RKO		Yes		Colorectal		/		Vemurafenib		Continuous						None		PIK3CA H1047R		77		PI3KCA/ Akt	
HT29_NRAS		Yes		Colorectal		/		Vemurafenib/cetuximab		NRAS overexpression						None		Upregulation of p-ERK		78			
Vaco432_NRAS				PDX from progression specimen		dMMR														78			

PDCL, patient-derived cell lines; PDX, patient-derived xenografts.

### 2.2 High-fidelity mouse models were used for testing combinatorial inhibitor therapy

**Figure 1:**
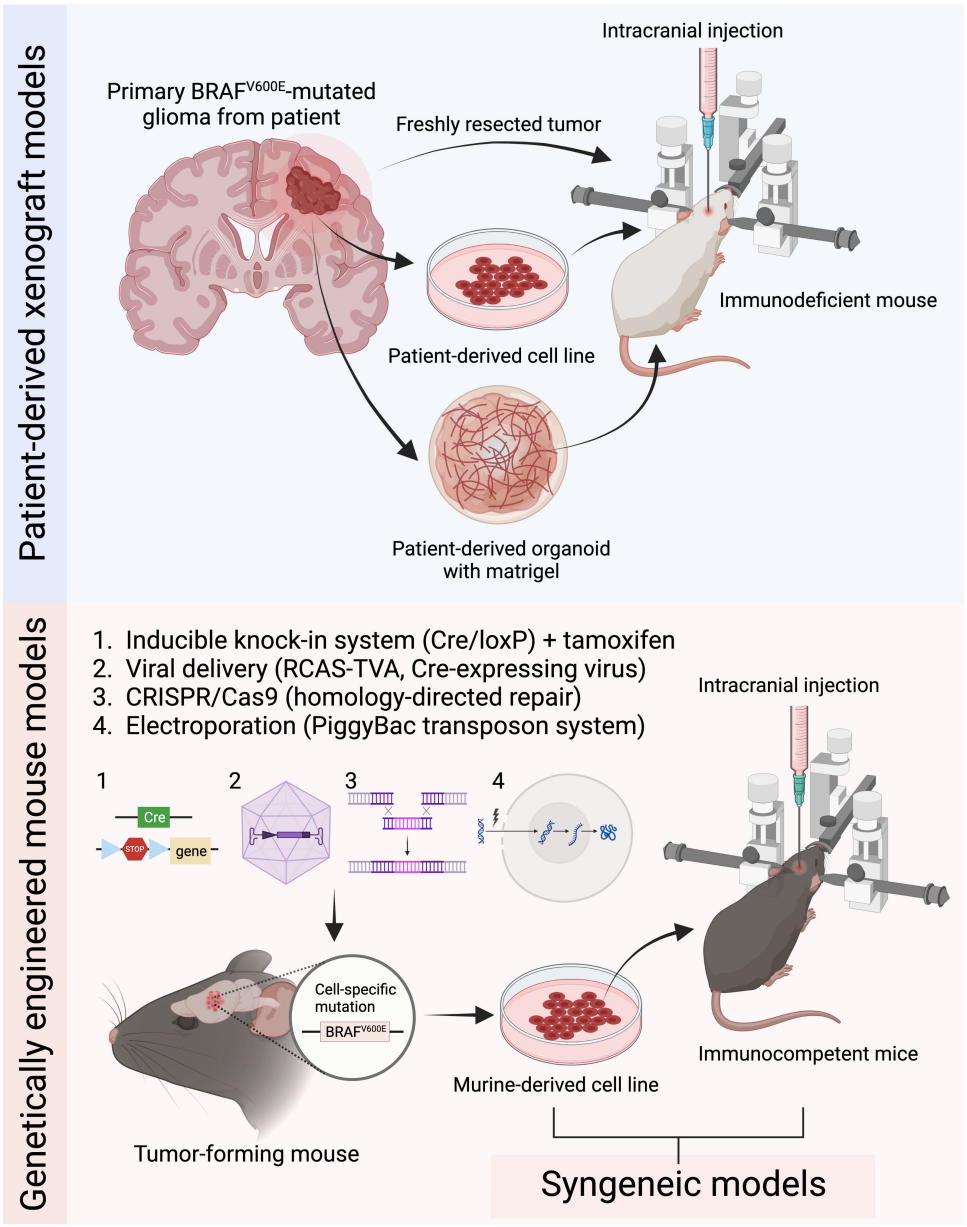
Current common preclinical mouse models for BRAF^V600E^ mutated gliomas. Patient-derived xenograft models are generated by intracranially implanting patient-derived tumor tissue, cells, or organoids into immunodeficient mice. Genetically engineered mouse models (GEMMs) induce tumor development through genetic manipulation using either the inducible knock-in system, virus-based strategies, CRISPR/Cas9 system, or *in utero* electroporation. Syngeneic models are established by implanting murine-derived tumor cell lines into immunocompetent mice (Figure created with BioRender.com).

Like studies in melanoma, where BRAF inhibitor monotherapy shows rapid but transient MAPK pathway inhibition, PLX4720 treatment in glioma cell lines (M-38, DBTRG-05MG and NMC-G1) did not suppress MAPK pathway signaling in a durable way. This prompted testing of vertical dual inhibition of MAPK pathway with BRAF and MEK inhibitors, an approach that has improved response rates in melanoma. Several studies indeed showed that MAPK signaling rebound in glioma can be overcome by adding MEK inhibition, including our work with a BRAF^V600E^ murine glioma cell line (2341) which were derived from an adenovirus-induced tumor in a Cre-inducible mouse model in which BRAF^V600E^ is expressed under the endogenous promoter, and hence under physiologic levels (22) (**Figure 1**). BRAF^V600E^ inhibitor monotherapy with dabrafenib of 2341 cells recapitulated the initial reduction followed by reactivation of MAPK signaling and accordingly 2341-derived orthotopic glioma transiently respond to BRAF^V600E^ inhibitor monotherapy with initial responses followed by continuous tumor growth. Combining BRAF and MEK inhibitors (dabrafenib+trametinib) suppressed MAPK pathway signaling in a more durable fashion (57, 80). Tumor regression from the combined dual MAPK pathway blockade with BRAF and MEK inhibitors was also confirmed in BT40 PDoX, which are described in greater detail below (81). In addition, combination therapy with PLX4720 + MEK inhibitor mirdametinib (PD0325901) (81) showed durable suppression of the MAPK pathway and this resulted in longer survival of animal subjects, further confirming our earlier data.

These studies combined prompted subsequent clinical evaluation of Vemurafenib against BRAF^V600E^-mutated gliomas (11) and more recently clinical use of BRAF and MEK inhibitor combination treatment in patients with BRAF^V600E^-mutated CNS tumors. Clinical responses showed an improvement in response rates with combination therapy over monotherapy (13). These data lead to the recent FDA approval of dabrafenib+trametinib for LGG and provide a paradigm for successful translation of preclinical data based on high-fidelity tumor models, including the 2341 model which expresses BRAF^V600E^ under the endogenous promoter like that in human tumors (57).

Continuous studies into novel combination therapies are important because of therapy resistance to vertical inhibition of MAPK signaling and cross-resistance to other BRAF/MEK inhibitors and BRAF/EGFR inhibitors in the clinic. Combinations investigated are of BRAF^V600E^ inhibitors with cdk4/6 inhibitor PD0332991 (22), PLK1 inhibitor tool compound BI2536 (82), radiation (83), MEK inhibitor AZD6224 + mTOR inhibitor everolimus (80), and EGFR inhibitor HKI-272 (84). These studies have used 2341 in addition to BRAF^V600E^ mutated CDKN2A^Null^ murine glioma cells derived from mice expressing BRAF^V600E^ using the CRE/Lox system under the nestin and GFAP-promoter, as well as established cancer cell lines.

Noteworthy, the 2341 orthotopic model can be implanted in fully immunocompetent mice allowing studies of immunomodulation and immunotherapy in the context of BRAF^V600E^-mutated gliomas. Using the 2341-BRAF^V600E^ model, we have recently demonstrated that BRAF/MEK inhibition alters the tumor immune infiltrate and sensitizes tumors against immune checkpoint blockade by anti-PD-L1 and anti-CTLA4 treatment, which resulted in better clinical outcomes in mice implanted with BRAF^V600E^ mutated high-grade gliomas (3).

In conclusion, preclinical data obtained using murine glioma models that faithfully recapitulate the human tumors’ genetics and phenotypes have high predictive capacity.

### 2.3 Patient-derived cell lines and media considerations

Conventional cancer cell lines are customarily grown as adherent monolayers on plastic dishes or laminin covered flasks in high-serum medium (typically supplemented with 10% FBS), for multiple generations. Although they are robust and easy to grow in this manner, the high-serum medium prompts cells to differentiate towards astrocytic phenotype, thus generating homogeneous cell cultures, which is unrepresentative of glioma’s biology. Moreover, the parental tumor of conventional cancer cell lines is frequently unknown and/or uncharacterized. As such, these models are useful for assay optimizations, including commercially available kits, imaging, monitoring morphological changes and optimizing antibody staining conditions. However, 2D monolayer architecture poorly represents patient-specific heterogeneity and affects tumor intrinsic features related to cell signaling, phenotype, cell-to-cell interactions, and drug response. The 2D cell arrangement generates inappropriate cell density and nutrient gradients, unphysiological oxygen levels, underrepresented spatial context, and artificial interactions with extracellular matrix and tumor microenvironment (85). In contrast, patient-derived cell lines (PDCLs) are initiated from tissue obtained during craniotomies, biopsies, and autopsies. Rapidly dissociated surgical samples are typically seeded in low-attachment cell culture flasks, and generate 3D spheres within a few days. Moreover, the culturing medium is serum-free and is commonly supplemented with growth factors (EGF and FGF-2), which perpetuates the stem cell phenotypes frequently observed in glioma cells and establishes robust glioma stem cell (GSC) cultures. Such patient-derived 3D spheroid cultures preserve features of the original tumor better than conventional 2D cancer cells lines (86) because they demonstrate higher proliferation and invasive capacity, high tolerance towards chemoradiation and sensitivity towards T- and NK-mediated cell killing (85, 87). This being said, PDCLs can be used as personalized platforms for drug testing and therapy tailoring (88), such as evaluating the benefit of single BRAFi vs combined with MEK/mTOR/EGFR inhibitors. A side-by-side comparison of patient-derived GBM cells has clearly demonstrated loss of malignant potential, decreased morphologic, and cell type heterogeneity, and divergence of expression profiles in cells grown in 2D differentiating conditions (high serum), versus 3D un-differentiating (serum-free) medium (89). Proliferation rates are frequent measures of drug activity in cell-based assays. These are significantly increased in adherent cells, while 3D spheroid cultures exhibit more stable doubling times even after multiple rounds of passaging. Furthermore, conventional 2D cultures are unreliable for functional analysis, as they undergo genetic drift over time, emphasizing the need for 3D spheroid models of CNS tumors (**Table 1**).

Our group recently generated a stable spheroid PDCL from a patient with a high-grade BRAF^V600E^ mutated tumor that also carried a TP53 and RB mutation (STN-10049) (3). This cell line was sensitive to the anti-proliferative effects of combined dabrafenib and trametinib treatment *in vitro*, although a marker for therapy resistant cells CD133 (82) was upregulated with dual MAPK inhibitor treatment, and the patient worsened rapidly despite aggressive chemotherapy and dabrafenib-trametinib treatment (3). Cells derived from the same patient’s tumor at autopsy showed decreased sensitivity to dabrafenib-trametinib, and increased expression of CD133, consistent with the patient’s lack of response and cancer progression on the drug. Thus, patient-derived cells derived from different timepoints (pre- and post-treatment) recapitulate longitudinal the patient response to therapy.

Several PDCLs representing various BRAF^V600E^-mutated CNS tumor subtypes have been generated and published by individual labs (see **Table 1** for a comprehensive list), and these lines are important novel tools available to investigators. Successful culturing and careful expansion of these models while monitoring for retention of driver alterations and genetic drift will ensure deep, comprehensive understanding of the biological and functional impact of BRAF^V600E^ mutation in glioma.

Molecular analyses of BRAF^V600E^-mutated glioma PDCLs are still scarce (**Table 1**, Other Alterations Reported), and the congruence of these cell lines with their tumor of origin has yet to be determined. Large scale initiatives such as the Human Cancer Model Initiative (HCMI) in the USA have been launched with the purpose of generating next-generation models, whereby “next-generation” refers to models being clinically annotated and are characterized molecularly and phenotypically for their resemblance to their parental tumor (see HCMI searchable catalogue). HCMI cell lines can be purchased from the American Type Culture Collection (ATCC). As of May 2023, no BRAF^V600E^-mutated CNS tumor cell lines have been reported in the HCMI searchable catalogue, but the efforts of the HCMI are ongoing, and expected to include more pediatric models soon.

In summary, PDCLs grown under 3D spheroid conditions are superior models of the human disease than conventional cancer cell lines and multiple individual labs. Novel BRAF^V600E^ PDCLs are continuously reported by individual investigators (**Table 1**) and larger scale model development initiatives and collectively these models will provide an important toolkit for robust preclinical and mechanistic studies of drug resistance and targetability.

### 2.4 Models for studying and overcoming therapy resistance

#### 2.4.1 Non-CNS cancer

Treatment with FDA-approved first-generation small molecule inhibitors of BRAF^V600E^, such as vemurafenib, dabrafenib, and encorafenib, showed clinical efficacy in some but not all glioma patients, raising the question how therapy resistance develops and how it can be overcome (90). Much data for therapy resistance mechanisms have been derived from BRAF^V600E^-mutated melanoma and to a lesser extent colorectal cancer PDCLs and cell lines (**Table 2**).

Preclinical studies in melanoma are the perfect example of how fundamental and translational findings improved clinical management of this disease. BRAF^V600E^ is prevalent in metastatic melanoma and novel strategies for BRAF and MEK inhibition are continuously developed and trialed to deliver long-term clinical response in patients. Combined BRAF and MEK inhibitors, as compared with BRAF inhibition alone, provided remarkable response rates in melanoma, delayed the emergence of resistance, and reduced toxic effects in patients with BRAF^V600E^-mutated melanoma (91, 92). Long-term survival analyses in metastatic melanoma showed that while one third of patients demonstrate long-lasting clinical benefit, the majority invariably progress with MAPK pathway inhibition alone (93). Resistance pathways discovered in melanoma can be divided broadly into three groups: RTK hyperactivation/overexpression, secondary MAPK/ERK mutations (ERK1/2 phosphorylation through NRAS mutations, paradoxical MAPK activation, AKT amplification/mutation, CRAF dimerization, BRAF amplification) and other alternative pathways (cyclin D1 induction, PTEN loss, BIM suppression) (73). Comparably, two independent studies of BRAF^V600E^-mutated melanoma cell lines (conventional and PDCLs) responded distinctively to PLX4032 (also known as RG7204 or RO5185426) (94, 95). Both studies show that treatment response differed among BRAF^V600E^ mutant cells. The diversity in sensitivity could be attributed to the additional co-occurring mutations contributing towards resistance (94) and/or differential expression levels of BRAF^V600E^ (95). Another study compared the sensitivity of A375 and WM115 melanoma cell lines to siRNA-mediated downregulation of BRAF^V600E^. WM115 are less sensitive to suppressed BRAF^V600E^ expression compared to A375 cells, but they were sensitive to PI3K inhibition. In both models dual inhibition of BRAF^V600E^ and PI3K signaling is more efficient in targeting melanoma cells than monotherapies (96). Altogether these results suggest that because of intra-tumoral heterogeneity, determining therapy solely on BRAF genomic status or single biomarkers seems insufficient. By these means, personalized approaches and rationally designing patient-specific therapies based on molecular signaling alterations evolving during disease progression might grant higher selectivity and longer-lasting effects. One great example of integration of network-based modeling and preclinical research is a study which set out to explore protein datasets (accessed at the Human Protein Atlas) of 353 BRAF^V600E^ and BRAF wildtype, and 372 thyroid carcinoma which were then used to identify patient-specific, co-expressed groups of onco-proteins in each tumor. These onco-proteins constitute a functional and targetable signaling network and a single tumor can harbor several distinct groups of signaling networks. Targeting all distinct “unbalanced” networks is necessary to disintegrate the altered signaling flux in the tumor. Once the 725 tumor samples were mapped based on the altered signaling pattern, Vasudevan et al. showed that their dataset is constituted of 138 different types of tumors, instead of just four (BRAF^V600E^ and BRAF wildtype and melanoma and thyroid cancer). They also showed that signaling patterns are distinct in some BRAF^V600E^ cancers, and that there are communalities with BRAF wildtype melanomas, which collectively confirmed that BRAF genomic status might be insufficient in assigning therapy. These findings were validated in melanoma cell lines (G361, A375 and A2058), which activate distinct signaling networks and required three different drug combinations to eliminate cell growth, despite carrying an identical BRAF^V600E^ mutation. As such, the predicted drug combinations were more efficient in tumor cell eradication, rather than monotherapies or dabrafenib + trametinib, often prescribed in the clinic (97).

Encouragingly, innovative approaches to study therapy resistance in cancer are rapidly emerging, including multi-omics approaches and computational modeling (98), and high-throughput CRISPR screening (99, 100). As such, targeted-exome, single-cell DNA and RNA-sequencing, ATAC- and ChiP-sequencing facilitated biological characterization, biomarker discovery and target identification among various cancer-treatment entities (98). Indeed, the activation of resistance mechanisms, as well as cell-cell interactions occurring in heterogeneous tumor samples/models can be mined by longitudinal sampling accompanied with classical or spatial multi-omics (101). However, the generation of such multidimensional, computationally heavy datasets requires competent teams of bioinformaticians and engines capable of dealing with complex molecular and pathological cues. Currently, there are three bioinformatic approaches available for computational resistance modeling. These include machine learning, network, gene co-expression modeling (network-based) and genome-scale metabolic modeling. Network-based modeling can identify gene co-expression patterns and mechanisms related to resistance. In addition, weighted network analysis indicates the level of significance of the co-expression link between gene pairs. Machine learning methods have a broader applicability regarding the type of datasets. Drug susceptibility and resistance can be predicted by logistic regression, random forest, and deep neural networks. Finally, genome-scale metabolic modeling informs on gene-protein interactions, to ultimately portray the global metabolic dynamics (98). In combination with the CRIPSR screening that enables unbiased evaluation of gene function by manipulating the target genes with CRISPR-Cas9 approach, various molecular mechanisms that confer drug resistance could be revealed (100).

A multi-centric study of BRAF inhibitor-resistant melanoma tissue samples, analyzed after disease progression, most detected secondary genomic alterations in the MAPK/ERK signaling pathway, BRAF copy number gains and BRAF alternative splicing, as mechanistic drivers of resistance. However, ~40% of samples did not provide a clear genetic cause of resistance (102). This set out research into unknown drivers and putative resistance mechanisms in preclinical models. *In vitro* models revealed secondary genetic resistance mechanisms, similarly, observed in patient tissue, whereby RTK expression was increased, through MAPK/ERK and/or PI3K/Akt signaling (**Table 2**).

In an *in vitro* study of resistance, a Luria – Delbrück fluctuation analysis was applied to generate two models of resistance propagated from single-cells, derived from PDCLs (WM989, WM983B): the first resistance condition was represented by a genetic “mutation”, heritable model, which harbors intrinsic capability to tolerate acute doses of vemurafenib (at 1μM). The second is a transient, non-heritable model, for which the resistant state is reversible and is achieved when a subpopulation of primed pre-resistant cells give rise to resistant colonies upon drug treatment, either through survival of resistant cell subpopulations, or epigenetic reprogramming of non-resistant cells. In addition, acquisition of secondary mutations can happen upon which the resistant state becomes irreversible (71). In both cases single cells were isolated, in order to minimize the effect of genetic heterogeneity and expanded for 7-8 divisions (generating approximately one million cells), after which the drug was added and colonies were counted. Thus, a large number of resistant colonies was detected in the heritable (genetic) model. In contrast, in the non-heritable model, there remained a high probability that any pre-resistant cell would result in a resistant colony. Untreated, intrinsically resistant colonies expressed high levels of *WNT5A, AXL, EGFR, PDGFRb*, *APCDD*, and *JUN.*

To examine temporal aspects of epigenetic resistance programs, cells were treated with one 1μM vemurafenib for four weeks, which resulted in an irreversible drug resistant state (71). After one week of vemurafenib treatment, pre-resistant cells expressed only a fraction of resistant genes (AXL, EGFR and NGFR) and after four weeks of treatment cells were fully reprogrammed and expressed the entire panel of resistance genes. ATAC-sequencing of transient cells uncovered broad cellular reprogramming by gain (TEAD, Jun/AP1) and losses (SOX10) of transcription factor occupancy. Once fully resistant, drug “holidays” (intermittent drug treatment) did not affect the resistant phenotype (71). In conclusion, epigenetic changes are sufficient to induce stable resistance to molecular targeted therapy.

Adding vemurafenib at progressively increased concentrations generated resistant sub-lines of M229, M249 and M238, through upregulated PDGFR-B and N-RAS (72). Interestingly, another study showed that vemurafenib-resistant cells develop *in vivo* and *in vitro* “drug addiction”, meaning that cells do not proliferate in the absence of the drug. In two PDX models, resistance was established 100 days after continuous administration of vemurafenib, while no resistant sub-clones emerged in the intermittently treated mice. Furthermore, cells derived from the resistant PDX models did not grow *in vitro*, unless the cell culture media was supplemented with small doses of vemurafenib (74). Similar observations were made in other *in vitro* studies of BRAF^V600E^-expressing cell lines (M288, SK-MEL28 and M14). Here, in absence of secondary mutations in NRAS, BRAF, KRAS, HRAS and MEK1, resistance emerged through BRAF amplification (74). These results suggest that dose and treatment schedule modulate therapy responses in patients.

Finally, the era of immunotherapy raised the question of the efficacy of combined BRAFi and immunomodulatory treatments in melanoma. This *in vitro* trial showed that vemurafenib-resistant sublines of M29 and M238 are cross-resistant to MART CTL and NK-mediated cell killing, pointing to intersecting apoptotic networks involving EGFR, PDGFRa, c-KIT, Met and IGFRb. In addition, researchers generated F5 CTL-resistant melanoma sub-lines through serial exposure to TCR-transgenic CD8+ T cells and found them to be cross-resistant to BRAFi. Interestingly, pretreatment of the resistant (vemurafenib and CTL) clones with HDACi (suberoylanidilide hydroxamic acid, SAHA) for 48 hours reversed their resistance to F5 CTL and NK-killing, but not to BRAFi. SAHA sensitized the cells by increasing the expression levels of proapoptotic regulators, caspases, TNF/TNFR family and death domain proteins (TNFSF10, TNFRSF10B, 11B), and reduction of antiapoptotic signaling (73). These *in vitro* data suggest that HDACi might overcome acquired dual resistance and immunosensitize BRAF^V600E^ mutated cells to CTL and NK-killing.

In addition to melanoma, colorectal cancer (CRC) cell lines are also used as representative BRAF^V600E^ disease models. In contrast to BRAF^V600E^ melanoma cell lines, CRC cell lines present higher activity of PI3K/AKT pathway and lower levels of phosphorylated MEK, ERK, RSK, cyclin-D1 and Myc (76). Upregulation of ERK/MAPK signaling pathway is a driver mechanism of CRC, whereby cancer progression is warranted by constitutive activation of RAS and BRAF. Driven by the successful inhibition of BRAF^V600E^ in melanoma, dabrafenib and vemurafenib were approved drugs for CRC treatment. However, ERK rebound in CRC is secured mainly by EGFR activation. Although, CRCs are largely inert to BRAFi monotherapy still, combined RAF and EGFR inhibition is shown to have some clinical benefit (78). However, even dual inhibition of RAF/EGFR in CRC can be overcome by wild-type NRAS amplification, which has been identified as a unique adaptive mechanism in CRC, but not in melanoma (78).

Resistant *in vitro* models commonly established from Colo205, HT-29, VACO432 and RKO cell lines and patient-derived xenografts of progression samples were shown to be sensitive to combined treatment of BRAFi and AKTi (PLX4720/vemurafenib and MK-2206) (76, 77). Tumor regression was also achieved by combining BRAF inhibitor BGB659 with EGFR inhibitor cetuximab in RAS - amplified, vemurafenib - resistant models (78).

#### 2.4.2 CNS tumors

We are only beginning to understand resistance mechanisms in CNS tumors (103), mainly due to the scarcity of resistance models. In a study of BRAFi cellular resistance, we have treated mice carrying BRAF^V600E^-mutated flank xenografts with BRAFi tool compound PLX4720 for almost a year. Cell isolates from these PDX have shown increased levels of CD133 and Nestin, which suggests upregulation of stem-like cells, but molecular mechanisms of resistance have not been analyzed (82). Patient-matched pairs of cells from before and after treatment are rarely obtained, but are useful tools for studying therapy resistance.

A novel promising therapeutic strategy emerged from using isogenic BRAF inhibitor sensitive (794, AM38, BT40) and resistant (794R, AM38R, BT76) cell lines and treating them with combinations of BRAF inhibitor (vemurafenib) and autophagy inhibitor (chloroquine). Importantly, clinical data support the use of autophagy inhibitors to overcome resistance to BRAF/MEK inhibition, suggesting successful translation of this approach. This study is impactful because it used several distinct models for BRAF^V600E^-mutated gliomas, including established cancer cell line AM38 as well as patient-derived cell lines (794, B78), a patient-derived xenograft (BT40), as well as patient-derived slice cultures (60) (**Table 1**). This study is an example of preclinical data derived from multiple models which were followed by rapid clinical responses in BRAF-inhibitor resistant patients. Consistent preclinical data from multiple models are thought to be of higher predictive value than those obtained from a single model, due to model-specific bias.

Another study used patient-derived glioma tissue from paired pre-/post- BRAF/MEK inhibitor treatment to identify treatment-related changes in gene alterations and expression using RNA and DNA sequencing. They performed functional validation of candidates and found that resistance mechanisms are varied and highly PDCL-dependent, further underscoring the importance of using multiple models for functional studies (104). Yet another study generated patient-derived cell lines and xenografts from primary tumors (YMG62P, YMG89P, NGT41P) and recurrent samples (cells collected from CSF: YMG62R, YMG89R, NGT41R) from patients relapsing after dabrafenib+trametinib treatment. Here, refractory cells showed sensitivity to HSP90 in combination to dabrafenib or trametinib treatment (67). Moreover, we have recently isolated tumor cells from a patient with BRAF^V600E^-mutated high-grade glioma, pre-/post- BRAF+MEK inhibitor combination therapy. We found that post-treatment samples exhibit resistance to BRAF/MEK inhibition, which is accompanied by elevated expression of markers associated with cancer stem cells, differentiation, and chemo- and radiation-therapy resistance, including CD133, in the post-treatment tumor (3).

**Figure 2:**
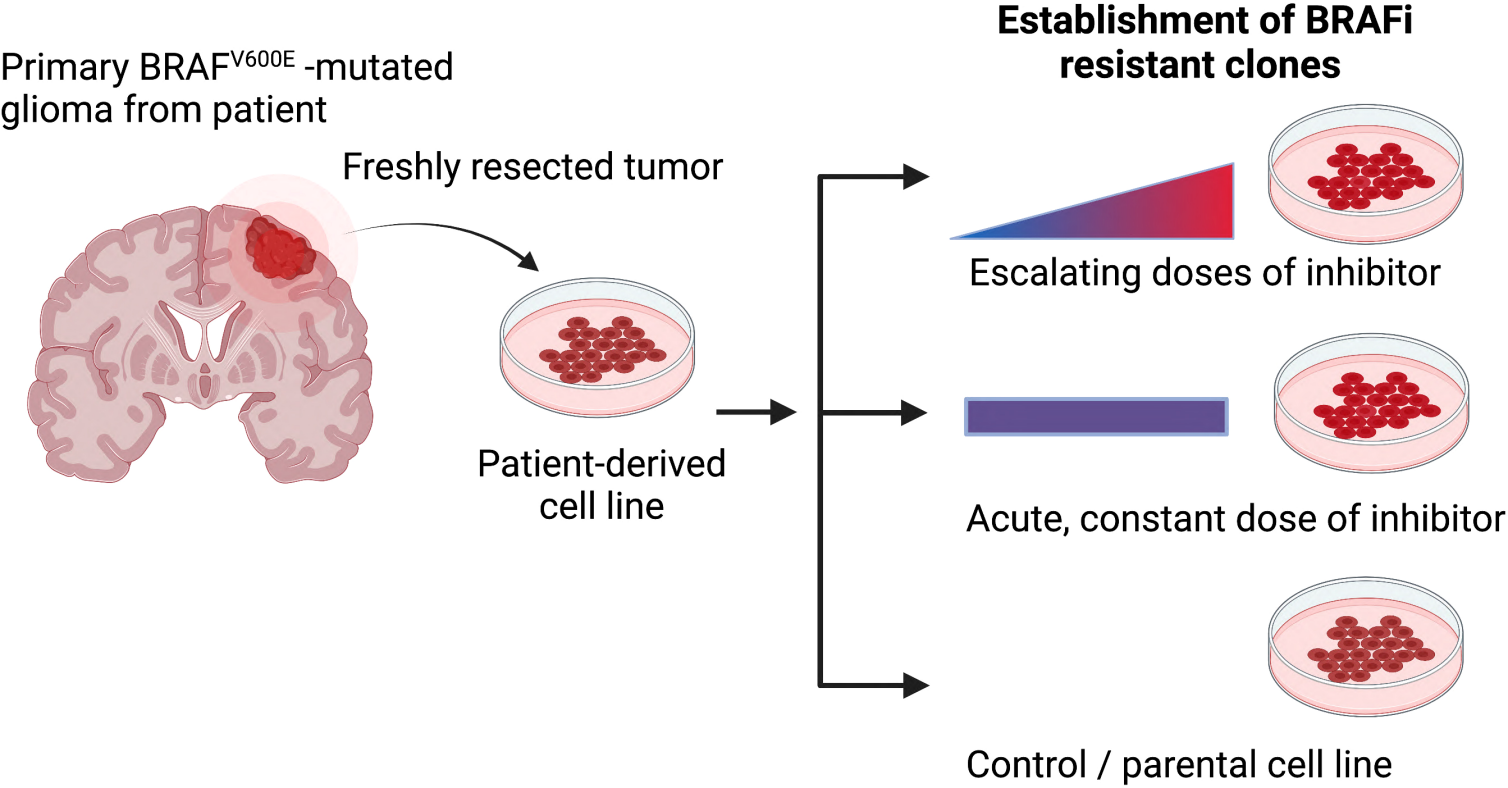
The most common methods for generating drug-resistant cancer cell lines. Primary patient-derived cell lines are treated either with escalating or constant doses of the relevant inhibitor over an extended period of time. Secondary, resistance associated mechanisms can be evaluated when comparing resistance and parental models (Figure created with BioRender.com).

Given the rarity of such patient-matched samples from before treatment and after treatment when resistance has formed, alternative approaches are needed, such as forcing cells *in vitro* to acquire resistance using chemical approaches. Chronic treatment with escalating doses of the chosen therapeutic agent or acute, constant doses of the inhibitor until refractory subclones overpopulate the cell culture are useful approaches to induce resistance (**Figure 2**). As such, adaptive, functional mutations were identified in vemurafenib resistant cell lines (AM38, MAF-794) (103). Moreover, CRISPR-mediated genome editing can be applied to studying drug resistance (intrinsic and acquired), through functional screens and candidate approaches (100).

In conclusion, validation of actionable molecules in resistant cells and preclinical studies of novel drug combinations that target these molecules using high-quality platforms are expected to overcome clinical limitations of BRAF/MEK inhibitors. Preclinical models provide unparalleled insights into intrinsic and acquired mechanisms of BRAF/MEK inhibitor resistance and models can be generated under various experimental conditions.

### 2.5 Patient-derived xenograft (PDX)-derived cell lines

Certain types of CNS tumors, including low-grade glioma, have a high failure rate for developing into patient-derived cell lines. Implanting surgical tissue directly into mice to generate patient-derived xenografts (PDX) and derive cell lines from PDX tissue is a valid alternative approach to culturing cell lines from surgical tissue directly. The PDX-to-cell line approach was used to generate a heterozygous BRAF^V600E^-mutated cell line BT-40 (63). BT-40 cells exhibited sensitivity to MAPK inhibitors selumetinib and trametinib (64, 105). A MAPK inhibitor library screen against BT-40 expressing a luciferase-based MAPK reporter assay revealed distinct sensitivities to single inhibitors, including next-generation RAF inhibitors, and synergistic effects of different inhibitor classes (106). BT-40 was also used to assess the clinical potential of ulixertinib, a first-in-class ERK inhibitor, and to test combinatorial effects with MEK inhibitors or BH3-mimetics (107).

However, it is unclear how well the low-grade parental tumor phenotype is preserved in BT-40 cells since they have been cultured in serum after passaging them as PDX. An additional caveat of patient-derived orthotopic xenografts (PDoX)-derived cell lines is potential contamination with mouse cells since they are hosted in mice brains; However, the BT-40 cell line had identical short tandem repeat analyses to the parental tumor and was free from mouse cells, as determined by LDH isozyme analyses. Whether BT-40 exhibits a gain of chromosome 7q identical to the xenograft has not been determined (105). In conclusion, PDX serve as an alternative source for cell lines and their ability to recapitulate the parental tumor needs to be monitored as it might change over time.

### 2.6 Limitations of existing *in vitro* models

There are clear limitations with existing models, the most prevalent limitation being that most of these models represent high-grade CNS tumors with combined BRAF^V600E^ expression and CDKN2A deletion; progression models that recapitulate progression from low grade to high grade tumors are not available. Combinations of BRAF^V600E^ with other rarer coinciding alterations are rarely represented. CNS tumors display marked inter-tumoral heterogeneity, which emphasizes the need for using multiple cell lines for each tumor class. Unfortunately, there are not enough cell lines available to the community to capture representative patient-to-patient differences. Moreover, despite the relative frequency and poor clinical outcome of BRAF^V600E^ mutant-brain metastases, there is only one published genetically engineered model for BRAF^V600E^ melanoma metastatic to the brain (53, 108), which severely hinders studies of the mechanisms underlying the metastatic process.

## 3 *In vivo* modeling

### 3.1 *In vivo* models for BRAF^V600E^ mutated neoplasms

To study the precise role of BRAF^V600E^ mutation in gliomagenesis *in vivo*, various mouse models of BRAF^V600E^-mutated glioma have been developed to better understand the complexity of tumor biology. Transgenic or genetically engineered mouse models (GEMMs), syngeneic models, patient-derived xenografts (PDX) in immunocompromised mice, and virus-induced models are commonly used to recapitulate certain biological features of primary patient BRAF-mutated tumors (**Table 3**). Although these models provide a valuable resource for studying how BRAF^V600E^ is involved in tumor development and recurrence following therapy, each model comes with its own benefits and limitations that need to be carefully considered.

**Table 3:** Existing mouse models of BRAF V600E-mutated gliomas.

Model type	Cell lines or virus	Host mice used	Age of mice	Tumor type	Injection site	Median survival	Cellular origin	Main findings	Reference	
PDX	IC-3635PXA cells with BRAF V600E expression and CDKN2A deficiency	NOD/SCID	ND	PXA	Cerebellum Cerebrum	150-170 days	ND	•Increased proliferation •Loss of GFAP and gain of Vimentin expression •Reactive gliosis •Perivascular metastasis •Xenograft cells with homozygous CDKN2A deletion shifted from disomy to trisomy chromsome 9	(109)
PDX	D645 V600E mutant	Athymic nude	6-8wks old	PXA	Striatum	25-50 days	ND	•Temozolomide, bevacizumab, CPT11 and sorafenib inhibited tumor growth in both V600E-mutant and V600E-non-mutant xenograft models. •MEK inhibition (cobimetinib) but not BRAF inhibition (vemurafenib) inhibited tumor growth	(110)
PDX	BT-40 NCH-MN-1 IC-3635	SCID SCID SCID	ND ND ND	ND ND PXA	ND ND ND	~21 days ~21 days ~28 days	ND ND ND	•Sensitivity of BRAF-mutated tumors to trametinib is due, in part, to MAPK-mediated regulation of TORC1 activity •Resistance to trametinib is driven by upregulated MAPK pathway and downregulated TORC1 pathway •Resistance to trametinib is tumor line-specific and drug-specific	(64)
PDX	DBTRG05-MG AM38	Athymic nude Athymic nude	5wks old 5wks old	Astrocytoma Astrocytoma	Striatum Striatum	~35-100 days ~20 days	ND ND	•DBTRG05-MG xenografts exhibited features including: invasion, analplastic tumor cell morphology, Nestin expression and high mitotic index •Combination therapy using BRAF inhibitor and CDK4/6-specific inhibitor to both cell lines inhibits tumor growth and extends animal survival	(22, 79)
Syngeneic	2341-luc cells with BRAF V600E expression and CDKN2A deficiency	FVB/N NSG	ND ND	Astrocytoma Astrocytoma	Corpus callosum Corpus callosum	58.5 days 45 days	ND	•Tumors co-express GFAP, Olig2, Ki67 and phospho-Erk •Combined BRAF and MEK inhibitor treatment prevented MAPK pathway reactivation and reduced tumor growth	(57)
GEMM	pCAG-Cre-IRES2-GFP or pCAG-Cre-GFP plasmid	Braflsl-V637E/+	E14.5	GG	IUE into the ventricle	–	NPs	•BRAFV600E mutations in neural progenitor cells during development lead to: epileptic seizures, which are often associated with GG and PXA neuronal abnormalities, increased numbers of astro- and oligodendroglia •BRAFV600E-induced epileptogenesis is mediated by RE1-silencing transcription factor (REST), a regulator of ion channels and neurotransmitter receptors associated with epilepsy	(111)	
Cre-lox system	CamK2-Cre-expressing adeno-associated virus	Braflsl-V637E/+; Rosa26lsl-tdTomato	P30	GG	Somatosensory cortex	–	NPs			
Virus-induced					(S1 cortex)					
GEMM Cre-lox system Virus-induced	Cre-expressing adenovirus	BRAFV600Efl/+ BRAFV600Efl/+Ink4a-arf-/-	60 day old 60 day old	Astrocytoma	Subventricular zone	– 70 days	ND	•Tumor formation in mice with concurrent heterozygous BRAFV600E expression and homozygous deletion of Ink4a-Arf upon Ad-Cre injection, but not in mice with BRAFV600E expression alone •Tumors exhibit strong immunoreactivity for Olig2, GFAP and Nestin •Tumors have impaired capacity to differentiate into neurons, OLs and astrocytes; •Tumors have high proliferative rate •Diffuse invasion throughout the cerebral hemisphere and white matter tracts	(22)
GEMM, syngeneic	Olig2-cre;BRAFV600Efl/+ hGFAP-cre;BRAFV600Efl/+ hGFAP-cre;BRAFV600Efl/+Ink4a-arf-/- Ad:cre;BRAFV600Efl/+Ink4a-arf-/-	SCID SCID SCID SCID	ND ND ND ND	Astrocytoma	Subventricular zone	ND ND 112 days 112 days	NPs	•No evidence of tumor formation in the brain of hGFAP-cre;BRAFV600Efl/+ mice. •These mice with BRAF activation alone survived until 3 wks. of age •Orthotopic injection of hGFAP-cre;BRAFV600Efl/+ cells or Olig2-cre;BRAFV600Efl/+ cells into SCID mice do not induce tumor formation	(22)
GEMM RCAS-TVA system virus-induced	RCASBP(A)BRAFV600E	Nestin-TVA/Ink4a/Arf-/-	Newborn	GBM	ND	ND	NPs	•BRAFV600E expression induces aggressive gliomas in mice when combined with Ink4a/Arf loss or activation of the Akt pathway •Tumors with BRAFV600E and Ink4a/Arf deletion do NOT express GFAP, S100 and Olig2, but strongly express Nestin show evidence of diffuse infiltration, aberrant vasculature and necrosis •No evidence of tumor formation in mice injected with BRAFV600E alone	(112)
GEMM RCAS-TVA system virus-induced	RCAS-BRAF VE kin (the V600E mutated BRAF kinase domain)	Nestin-TVA	Neonatal	PA	Hemispheres Brainstem	ND	NPs	•Expression of BRAF VE kin induces tumor formation with strong immunoreactivity for GFAP and weak or negative immunoreactivity for Nestin •Strong phospho-Erk and Ki67 positivity	(113)
GEMM piggyBac transposon system	pGlast-pBase or pNestin-pBase transpose helper plasmids and pBCAG-Flag-BRAFV600E or pBCAG-Flag-BRAFwt donor plasmids	CD1	E14	–	IUE into S1 cortex	–	NPs	•BRAFV600E in neural progenitor cells results in hyperexcitable pyramidal neurons •BRAFV600E disrupts migration of pyramidal neurons destined for upper cortical layers	(114)
GEMM piggyBac transposon system	pCMV-pBase transpose helper plasmid and PB-CAG-BRAFV600E, or PB-CAG-BRAFV600E and -pAkt, or PB-CAG-BRAFV600E, -pAkt and -Cre donor plasmids	CD1 Trp53-/- with a CD1 background	E14 E14	PLNTY GG aGG	IUE into the ventricle IUE into the ventricle IUE into the ventricle	ND ND ND	Glioneural progenitors	•1-hit model: BRAFV600E alone induces tumor formation with oligodendroglial and PLNTY-like features; positive immunoreactivity for MAP2, Olig2 and CD34 •2-hit model: BRAFV600E and Akt activation leads to neoplastic tumor growth, exhibit substantial neuronal activity and epileptogenic propensity; a glioneuronal phenotype immunoreactive for MAP2, GFAP, NeuN and Olig2; tumors that recapitulate GG-like features •3-hit model: BRAFV600E/pAkt/Trp53-loss tumors exhibit glioneuronal clonality with aGG-like features	(115)
GEMM RCAS-TVA system CRISPR-Cas9 system	Plasmid carrying the BRAF gRNA and the BRAFV637E Homology-Directed-Repair donor was cloned into a lentiviral vector and transduced into the p53-null TVA-Cas9 neural stem cells	NOD/SCID	4-5wks old	ND	ND	66 days	NPs	•BRAF mutant/p53-null tumors show strong immunoreactivity for Ki67, Olig2 and Nestin, and elevated MAPK kinase activity as revealed by upregulated phospho-Erk •Resistance to dabrafenib monotherapy is driven by the upregulation of the MAPK signaling pathway	(116)

PDX: Patient-derived xenografts; GEMM: Genetically modified mouse models; ND: Not determined; PXA: pleomorphic xanthoastrocytoma; GG: ganglioglioma; GBM: glioblastoma multiforme; PA: pilocytic astrocytoma; PLNTY: polymorphous low-grade neuroepithelial tumors of the young; aGG: anaplastic GGs; IUE: in utero electroporation; NPs: neural progenitors.

### 3.2 Patient-derived xenograft models

These models involve the implantation of tissue into the flank and dissociated cells into the brain to obtain orthotopic xenografts (PDoX). Although the growth of PDoX is harder to follow due to their inaccessibility in the brain, they provide a more faithful representation of the tumor microenvironment and invasiveness of CNS tumors than flank tumors. Severe combined immunodeficient (SCID), non-obese/diabetic (NOD)/SCID, athymic nude, and NSG mice are typical hosts for PDX development, due to their impaired immune systems, which facilitates achieving graft survival without risk of short-term rejection. Although these models can mimic certain features of the original human tumors, such as genetic and molecular heterogeneity, the biological aspect of tumor initiation and growth cannot be studied. Moreover, they do not fully capture the immune system’s role in controlling glioma growth and progression, deeming them unsuitable for studying tumor immunology and evaluating immunotherapy response.

Establishing PDX models for BRAF^V600E^-mutated gliomas can also be time-consuming with a low success rate of engraftment (109) depending on the availability of patient tumor tissue with several factors taken into consideration, such as how the tissue is collected and stored. Several PDX biobanks have been established by individual groups and institutions, including PDX panels from GBM (117), and some specifically for pediatric brain tumors (118, 119). BRAF missense mutations have been identified in these PDX by molecular profiling, but due to the relative scarcity of BRAF^V600E^ altered CNS tumors amongst brain tumor patients, PDX for these tumors are rare. A PDX biobank specifically focused on BRAF^V600E^-mutated gliomas is currently lacking due to the scarcity of patient material, and the difficulty in retrieving complete patients’ clinical data, pathologies, gene expression profiles, and drug responsiveness (64). However, there is great potential for this to be an authoritative resource for developing new treatments and personalized medicine approaches.

### 3.3 Syngeneic murine models

These models are created by implanting an established tumor cell line derived from the same species as the host animal. The 2341 cell line of BRAF^V600E^-mutated and CDKN2A-deleted glioma injected into both immunodeficient (NSG) and immunocompetent (FVB/N) mice have revealed successful engraftment and has been critical for the preclinical assessment of experimental therapeutics (57). The use of syngeneic models of BRAF^V600E^-mutated glioma enables the study of the immune microenvironment associated with the tumor, and the testing of immunotherapies (3). However, syngeneic models may have limited translational potential since they do not fully recapitulate the biological and clinical features of human gliomas. Species-dependent differences in tumor microenvironment, immune response, tumor heterogeneity and therapeutic response are important factors that need to be taken into account. Multiple models generated from syngeneic mouse tumor lines for a particular type of glioma such as glioblastomas could also be variable in immune phenotypes among tumors, translating these findings into effective immunotherapeutic strategies for human glioma patients could be complex and warrants further research and validation (120). In addition, these models may exhibit inconsistent tumor growth rates, due to a functional immune response in host mice, which may affect the reliability of experimental results and require a large study cohort.

### 3.4 Inducible modeling systems

Knock-in GEMMs for BRAF^V600E^-mutated gliomas are alternative models that involve conditional and inducible systems such as the Cre-loxP system to allow the expression of BRAF^V600E^ under the control of the endogenous promoter and enhancer sequences in a cell-specific or time-specific manner upon the activation of Cre recombinase (57, 121), which can be induced by delivering either tamoxifen or Cre-expressing viruses such as adeno-associated viruses and adenoviruses (22, 111). The use of the inducible Cre-loxP system combined with knock-in GEMMs has provided valuable tools for understanding the effects of BRAF^V600E^ mutation in tumor progression in the context of an intact immune system and microenvironment, and for developing potential therapies that specifically target these mutations.

Another type of GEMM for BRAF^V600E^-mutated gliomas is the replication-competent avian sarcoma-leukosis retrovirus and the corresponding avian leukosis virus A (RCAS-TVA) system, which allows for somatic gene delivery to cells that are engineered to express cell surface receptor TVA using a cell type-specific promoter such as Nestin, which is a common marker for neural stem cells (112, 113). This enables investigation of the tumor cell of origin after BRAF^V600E^ mutation, as well as the role of BRAF in glioma development.

Transposon-based GEMMs have been developed for studying glioma development. These models include the Sleeping Beauty transposon system and the PiggyBac transposon system by which a transposon containing a mutation is integrated into the genomes of targeted cells of interest, resulting in the development of malignant tumors in the brain (122, 123). Specifically, BRAF^V600E^ mutation in neural progenitor cells induced by the binary PiggyBac transposase system has revealed the formation of GG-like tumors only in concert with the activation of Akt/mTOR signaling (115), as well as a hyperexcitable phenotype in neocortical pyramidal neurons with increased neuronal firing frequencies (114). This is also supported by another study showing a causal relationship between BRAF^V600E^ mutations and epileptic seizures, which are often associated with GG and PXA (111) (**Table 3**).

### 3.5 Challenges of modeling BRAF^V600E^ mutated gliomas *in vivo*

The inter-tumoral heterogeneity of BRAF^V600E^ mutated gliomas provides an additional modeling challenge. PXA express reticulin fibers and are therefore thought to originate from transformed subpial fibrous astrocytes (124), whereas GG consisting of mixed neuronal and glial components points to a neural stem cell origin. Since there is an ongoing debate over the cell of origin for gliomas with Nestin-expressing neural stem and progenitor cells (NSCs) and oligodendrocyte progenitor cells (OPCs) being the two major candidates to transform into tumor cells and to shape tumor heterogeneity, it is important to characterize the relative contribution of each population to glioma initiation and progression in the context of BRAF^V600E^ mutation.

Moreover, no studies have yet been conducted to determine the OPC nature of BRAF-mutated tumors using either the RCAS-TVA system or the Cre-lox system with OPC-specific promoters such as PDGFRα or NG2 (**Table 3**). This is particularly important since BRAF^V600E^ expression in Ink4a/Arf-deleted cells has been shown to increase the proportion of proliferative glial progenitor cells in adult mice *in vivo*, disrupt asymmetric cell division of OPCs, and increase OPC frequency *in vitro* at the expense of mature oligodendrocytes and astrocytes, respectively, without altering NSC frequency (3). These data suggest that OPCs are more sensitive to cellular transformation by concurrent BRAF^V600E^ expression and Ink4a/Arf loss than NSCs. These two alterations have yet to be combined in transgenic mouse models with OPC type-specific expression, to address whether OPCs can indeed be the origin of BRAF^V600E^ mutated tumors *in vivo*. Exploring whether BRAF^V600E^ mutation can also occur in mature astrocytes or oligodendrocytes that de-differentiate into stem or progenitor cells with tumorigenic potential would be intriguing to explore, since both astrocytes and neurons have been shown to function as the cells of origin for gliomas via dedifferentiation (125).

Nevertheless, the cellular origin of BRAF-mutated gliomas remains underappreciated, and further studies are required to understand the cellular and molecular mechanisms underlying the development of these tumors. Distinct cellular origins are just one potential explanation for the phenotypic diversity of BRAF^V600E^-mutated tumors; alternative explanations such as microenvironmental diversity, and distinct neurodevelopmental origin amongst the tumors with distinct age presentation are important underlying factors to be considered.

## 4 Challenges for establishing high-fidelity BRAF^V600E^ tumor models

### 4.1 The challenges of developing models for low-grade gliomas

Establishing robust *in vitro* models that are representative of low-grade CNS tumors, incuding BRAF^V600E^ -mutated CNS tumors is inherently challenging. Low grade tumors are notoriously difficult to model *in vitro* and *in vivo*, likely due to tumor cell differentiation states, slow growth senescence, and microenvironmental signals in culture media that favor outgrowth of normal brain cells. Indeed, certain copy number alterations have been associated with robust *in vitro* growth, including gain of chromosome 7p, and loss of chromosome 10q. Loss of PTEN (126) and CDKN2A loci (127) have been positively associated with the establishment of PDCLs. While high-grade tumors tend to have a higher frequency of establishing cell lines, it is challenging to maintain the phenotypic intra-tumoral heterogeneity that is so characteristic of high-grade CNS tumors; in addition, certain genetic alterations (ATRX, IDH1, and hTert) tend to get lost in cultured cells presumably due to negative selective pressure from culture conditions (128). Recent studies have successfully approached generating adult LGG marked by IDH1 alterations by picking slow-growing tumor cells and separating them from fast-growing normal fibroblasts, that typically outgrow from mixed patient-derived cultures (127). Moreover, patient-derived organoid cultures produced faithful *in vitro* models for adult IDH1-mutated LGG (129). Most *in vitro* BRAF^V600E^-mutated CNS tumor models created thus far are high-grade models, including 2341, AM38, DBTRG-M05, STN-10049. Encouragingly, some models for LGG are also reported, including BT-40 and IC3635-PXA (see **Tables 1**, **3**). Care must be taken that these models retain their low-grade features mentioned above since it is well-known that tumor cells can spontaneously become more aggressive when passaged *in vitro* over time.

In conclusion, attempts to generate *in vitro* models for low-grade CNS tumors have been hampered by negative selection against tumor cells in culture and phenotypic drift to a high-grade tumor over time. Recent studies from adult tumor types have provided new guidance for generating urgently needed robust *in vitro* models of pediatric CNS tumors in general and BRAF^V600E^ pLGG in particular.

### 4.2 Developing models for tumor progression from low to high grade is challenging

**Figure 3:**
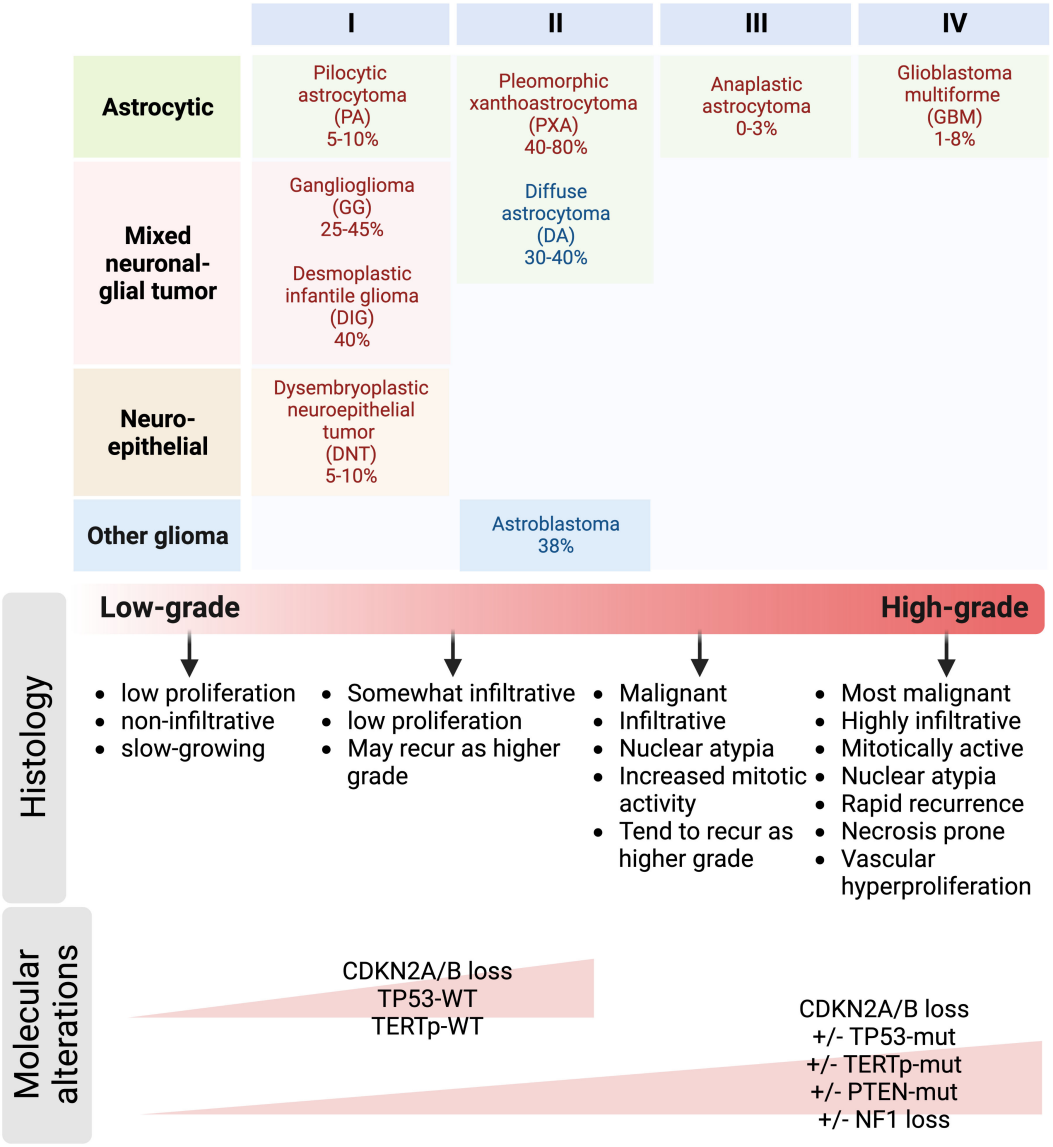
Prevalence of BRAF^V600E^ mutation in pediatric and adult gliomas. Gliomas are classified into tumor subtypes and WHO grades based on both histological and molecular features. Grade 1 and 2 gliomas are often referred to as low-grade gliomas, whereas grade 3 and 4 gliomas are referred to as high-grade and malignant gliomas. Histological characteristics such as nuclear atypia, mitotic activity, necrosis, and endothelial proliferation are often used to differentiate low-grade from high-grade tumors. Key molecular alterations co-occurring with BRAF^V600E^ mutation include CDKN2A/B deletion, mutations in TP53, TERT gene promoter, and PTEN; and NF1 loss can also help distinguish low-grade from high-grade gliomas.

Adult LGG commonly evolve into higher grade tumors, while malignant transformation occurs less frequently in pLGG, with BRAF^V600E^-mutated pLGG being an important exception (5, 18, 20, 27). Most *in vitro* and *in vivo* models in the field of neuro-oncology are for HGG, and genetically engineered mouse models recapitulating BRAF^V600E^-mutated LGG and their progression to high-grade are lacking, due to the slow-growing nature of these tumors. Thus, the biological mechanisms underlying the onset of LGG formation remains poorly understood and molecular and cellular mechanisms for progression to a more aggressive clinical course of BRAF^V600E^-mutated gliomas are unknown. To create an ideal preclinical model for BRAF^V600E^-mutated LGG that represents the complex and dynamic tumor microenvironment of malignant transformation, the grading and classification of gliomas need to be recapitulated as closely as possible in animal models. As indicated in **Figure 3**, the WHO grading system consisting of cellular density, nuclear atypia, mitosis, necrosis, and endothelial proliferation is commonly used to evaluate malignancy based on the tumor’s histopathological appearance with high grade tumors having more aggressive microscopic features than low grade tumors (130). High grade tumors often have increased nuclear pleomorphism with variability in shape and size, as well as elongated, large, and hyperchromatic nuclei. They also have increased cellularity, mitotic activity, necrosis, and vascular hyperproliferation. High grade tumors have a higher Ki67 proliferation index indicative of more aggressive tumor growth and they tend to exhibit high immunoreactivity for stem cell markers such as CD133, Nestin, Sox2 and Olig2. However, functional roles and contribution to tumor behavior during progression and treatment response of stem cells in LGG are still being investigated, and therefore the clinical significance of stem cell marker expression in glioma progression remains unclear. Asymmetric divisions of glioma cells regulate adult tumor cell fate and perpetuate a stem-like phenotype and contribute to tumor expansion (82, 131, 132). Regulation of asymmetric division in LGG has yet to be determined.

To further improve the classification of gliomas due to the limitations of the histological grading system such as subjectivity with inter-observer variabilities and intra-tumor heterogeneity (133), molecular markers are often integrated into a more clinically relevant grading scheme. Some alterations such as CDKN2A/B loss, PTEN, EGFR, NF1, and TERT promoter mutations have been shown to be associated with very poor clinical outcomes in patients with BRAF-altered gliomas (17). However, some studies reported conflicting results regarding the prognostic value of some molecular markers (133), emphasizing the importance of understanding the molecular mechanisms underlying glioma progression. This poses another challenge in generating preclinical models of LGG due to the difficulty in recapitulating the widespread genomic and epigenomic effects of BRAF alterations. Despite the mechanistic challenges in creating an ideal mouse model of BRAF^V600E^-mutated LGG, it is feasible using the genetic engineering techniques with the following factors considered: *1)* cell type-specific BRAF^V600E^ mutation induced by either Cre-loxP or RCAS-TVA system, *2)* selection of the appropriate genetic background of the mouse which can influence the development and progression of tumors, *3)* monitoring of tumor progression by MRI to detect the presence and growth of tumors, *4)* validation of the model by comparing its histopathological and molecular characteristics to human BRAF-mutated LGG, and *5)* the validated mouse model can be used for preclinical research to test drugs to better understand the underlying biological mechanisms of BRAF-mutated LGG and this will pave the way for the identification of therapeutic targets to block tumor progression.

In conclusion, LGG models are needed to better understand the role for stem cells and asymmetric division in tumor formation and progression. Due to the complexity of generating a mouse model that accurately recapitulates the genetic background, intra-tumoral heterogeneity, and tumor microenvironment that all closely resemble those of human tumors, multiple models may need to be generated and evaluated before an ideal model is established.

### 4.3 Loss of oncogenic alterations *in vitro*

Recurring oncogenic alterations are difficult to maintain *in vitro*, including IDH1 mutations, which are reportedly frequently lost in PDCLs grown under standard serum-containing conditions (134). One study recently reported successfully generating several IDH1 mutated glioma cell lines by growing them under serum-free conditions and selecting cells manually based on their tumor cell morphology and separating them physically from the non-tumor associated cells that are IDH-wildtype (127). Such labor-intensive approaches might not be necessary when attempting to culture BRAF^V600E^-mutated PDCLs, since BRAF^V600E^ expression was maintained in culture and upon xenografting *in vivo*, suggesting that cells remain dependent on activated MAPK pathway signaling (3). Notably, one study reports an increase in allelic frequency (from 28% in the patient tumor) to 70% in PDoX, and up to 69% in PDoX-derived cell lines, indicating that BRAF^V600E^ plays an important role in tumor progression (109). In conclusion, BRAF^V600E^ expression might be required for tumor cell growth and therefore maintained in culture. Whether BRAF^V600E^ expression provides a selective disadvantage or advantage in cultured cells has yet to be systematically explored.

### 4.4 Modeling an intact immune system and microenvironment

#### 4.4.1 Syngeneic and humanized mouse models

Given the key physiological and pathological processes in mice differ substantially from those in humans and most of the traditional mouse models are immunocompromised, humanized mouse models engineered to carry human genes, cells or tissues (usually patient-derived or human cell line-derived xenografts) have been developed, and especially those mice with functional human immune components are an attractive alternative for understanding human cancer immunology and for testing immunotherapies, therefore leveraging their value in translation research. These humanized mouse models involve stable engraftment of either human CD34+ hematopoietic stem cells that produce multi-lineage human immune cells or human peripheral blood monocyte cells, and when combined with orthotopic implantation of patient-derived or human cell line-derived xenografts, human-specific biological processes during tumorigenesis in an immunological context can be studied. In addition, knock-in humanized mouse models are also available to better understand the anti-tumor response of immune checkpoint inhibitors (ICIs) targeting human-specific molecules in fully immunocompetent mice.

For example, since the combination of BRAF and MEK inhibition with immune checkpoint blockade by anti-PD-L1 and anti-CTLA4 treatment exhibited clinical benefit in mice implanted with BRAF^V600E^-mutated high-grade gliomas (3), the generation and characterization of knock-in humanized mouse models that express both the BRAF^V600E^ mutation and human PD-1 and CTLA-4 molecules would allow researchers to test the efficacy of combination therapies targeting both the BRAF^V600E^ mutation and immune checkpoint molecules in a more human-like setting. Indeed, the double-humanized PD-1 and CLTA-4 knock-in mouse model has been validated (135); in conjunction with mouse models of BRAF^V600E^ mutated glioma, this enables the studies of molecular and immunological mechanisms of ICI response to glioma, as well as the studies of tumor-immune interactions.

## 5 A critical comparison of existing mouse models of BRAF^V600E^-mutated glioma

Existing mouse models of BRAF^V600E^-mutated glioma in the literature are summarized in **Table 1**. Different mouse models have shown varying median survival times, ranging from 45-170 days (**Table 3**) (22, 109). This suggests that the effects of BRAF^V600E^ expression are modulated by additional factors that impact tumor formation.

With forced full-length expression of BRAF^V600E^ using either RCAS virus or the Cre/Lox system, respectively, robust tumors were only formed when combined with other oncogenic mutations such as CDKN2A deletion, Akt overexpression, and/or p53 deficiency (22, 112, 115). Tumors with BRAF^V600E^ expression and CDKN2A deficiency remained in an undifferentiated state with one study using the Cre/Lox system showing strong immunoreactivity for GFAP, Olig2, and Nestin, whereas in another study using the RCAS system, tumors with the same genetic mutations exhibited positive immunoreactivity for Nestin, but not GFAP and Olig2 (22, 112) (**Table 3**). In addition, the former study revealed that BRAF^V600E^ CDKN2A^Null^ tumors tend to proliferate and invade throughout the cerebral hemisphere and white matter tracts, while the latter study did not show evidence of diffuse infiltration of tumor cells. These histopathological differences could be explained by differences in expression levels, since the CRE/Lox-driven model drives BRAF^V600E^ expression from the endogenous promoter, while the RCAS model uses an unphysiological promoter. Despite these differences both studies consistently demonstrated that BRAF^V600E^ expression requires additional mutations to induce formation of high-grade lesions.

Importantly, overexpression of a truncated BRAF kinase domain carrying the V600E mutation (BRAF VE kin) but lacking the autoinhibitory domain in mice by itself can induce slow growth of tumors resembling human PA (113). This was attributed to the increased protein abundance of phosphorylated Erk in BRAF VE kin, resulting in stronger MAPK activation that was sufficient to promote tumorigenicity without further oncogenic mutations. Cases-Cunillera et al. later used this truncated BRAF^V600E^-containing kinase domain in the piggyBac transposon system to demonstrate that this truncated variant alone indeed induced tumors, but with oligodendroglial and PLNTY-like features.

In conclusion, the phenotypic spectrum of BRAF^V600E^-positive mouse CNS tumors is influenced by the type of mouse model used. A major modulating factor is the regulation of BRAF^V600E^ expression and whether it is driven by an exogenous or the endogenous promoter, which presumably causes variance in expression levels of the mutant kinase leading to differential signaling strengths. The presence of the BRAF autoinhibitory domain and additional oncogenic mutation(s) and the presence or absence of negative regulators of BRAF (e.g., Sred), and negative feedback loops are additional potentially modulatory factors of tumor phenotypes (**Table 3**).

## 6 Enhancing mouse models of BRAF^V600E^ mutated glioma for preclinical studies

Since mouse models of glioma provide an important platform for testing potential therapies and investigating pathological mechanisms underlying tumorigenesis, the usefulness of these models for preclinical studies can be further enhanced by combining them with a variety of advanced technologies and tools, which are briefly reviewed below.

### 6.1 Non-invasive imaging techniques

One such technology is non-invasive imaging techniques including magnetic resonance imaging (MRI) and bioluminescence imaging (BLI), which enable the visualization and monitoring of tumor growth and assessment of tumor response to treatments in mice. MRI allows the identification of tumor size, shape, location, and invasion into surrounding tissues, whereas easily accessible and commonly used BLI can only monitor the growth of tumor cells that have been engineered to express luciferase, an enzyme that produces light that can be measured throughout BLI when the luciferin substrate is given. However, in contrast to MRI, there are some limitations of BLI that need to be seriously considered: *1)* it has limited penetration depth of light so tumor cells located deep in the brain could be hard to detect, *2)* it has a limited dynamic range, making it difficult to accurately measure tumor growth changes; the bioluminescence signal measured from tumor cells can sometimes saturate at high cell densities at later stages, and *3)* unreliable quantification of BLI signal due to variations in the expression of the luciferase reporter gene in tumor cells. Indeed, a recently published study has demonstrated that the discrepancy in the evaluation of tumor growth by BLI and MRI was attributed to the instability in luciferase expression in the viral construct (136). In contrast, MRI allows for more accurate detection and measurement of tumor size and location in the brain, enabling researchers to perform longitudinal studies, where the tumor growth and progression can be monitored by analyzing changes in the tumor size and shape over time. As a result, BLI should ideally be used in conjunction with MRI if feasible at several time points to obtain complementary information for the accurate interpretive analysis of tumor growth and response to therapy in preclinical studies.

In terms of the assessment of the response to BRAF/MEK inhibitor combination therapy, biochemical analyses for evaluating kinase inhibitor activity in the brain are a time-consuming process that often involves tissue dissection, which may lead to the disruption of the activity being measured. Novel kinase-modulated bioluminescence indicators enable noninvasive imaging of signaling strength and could help tracking the activity of target kinases in response to targeted drug treatment (137). Such approaches represent valuable tools for improving drug discovery against BRAF^V600E^ mutated glioma and they might help with developing biomarkers which are urgently needed to stratify patients and identify those that will need additional treatments in addition to BRAFi+MEKi combination therapy.

### 6.2 Behavioral consequences of BRAF^V600E^ mutated gliomagenesis

Accumulating evidence suggests that BRAF^V600E^-mutated gliomas are associated with an increased risk of epilepsy and seizure, which can have a range of behavioral consequences based on their frequency and severity. As indicated in **Table 3**, some studies using mouse models revealed that BRAF^V600E^ mutation arising from progenitor cells during brain development leads to the acquisition of intrinsic epileptogenic properties in neuronal lineage cells and tumorigenic properties in glial lineage cells. This somatic mutation contributes to epileptogenesis by upregulating the expression of the RE1-silencing transcription factor which can repress a subset of genes coded for ion channels and receptors that are crucial to neuronal function (111, 138). It has also been demonstrated that BRAF^V600E^ mutation in neural progenitor cells results in a hyperexcitable neuronal phenotype (114). Since the BRAF^V600E^ mutation has been strongly linked to changes in neuronal excitability, it would be intriguing to determine whether there is a cognitive impairment as a result of glioma-associated epilepsy induced by BRAF^V600E^ mutation. Nevertheless, further studies are required to better understand the relationship between BRAF^V600E^ -mutated gliomas and epilepsy and the underlying mechanisms and potential therapeutic implications. Currently, behavioral assessment in mouse models of BRAF^V600E^-mutated glioma that involves testing of motor and cognitive functions is lacking and thus is important to explore. This will also validate whether these preclinical models can mimic human patients’ cognitive decline and epileptogenic properties.

### 6.3 Single-cell sequencing technologies

Intra-tumoral heterogeneity has been strongly implicated in affecting treatment response and conferring resistance in glioma with the underlying cellular and molecular mechanisms remaining fully unknown (139). Furthermore, the molecular responses of high versus low grade tumor cells carrying the BRAF^V600E^ mutation to BRAF and MEK inhibition have not been investigated in detail and thus the differential responses in patients with LGG and HGG to these therapies are poorly understood (13). Recent advances in single-cell sequencing technology would help address these questions since it allows for the integrative analysis of the genome, transcriptome, epigenome, proteome, and/or metabolome-based characterization of the individual cell among cancer cells within a tumor. Single-cell transcriptomic analysis of A375 (BRAF^V600E^ mutant) melanoma cell line described functional and phenotypic changes during development of drug resistance towards targeted therapy. Overall, four different treatment conditions were applied to the A375 cells (Vemurafenib monotherapy, V + Cobimetinib, V + trametinib, and untreated control). ScRNAseq revealed that the initial events of mono-drug resistance include loss of differentiated antigen presenting and neural crest-like cells and enrichment of undifferentiated, heterogeneous, highly proliferative stromal-like cells. Double-agent resistance featured variable amounts of dedifferentiated stromal cells because of heterogeneous effects of therapies on cellular states. In addition, specific survival machinery related to MAPK, p53 and PI3K/Akt reactivation and pluripotency induce distinctive cell populations (140).

Clonal barcoding is a powerful single-cell technique that allows researchers to track clonal evolution trajectories of tumor cells and to identify genetic changes and chromosomal aberrations that drive tumor growth and progression, or tumor cell resistance over time. A recently developed “CAPTURE” single-cell barcoding approach has been utilized in combination with an in-depth multi-omics analysis to reveal the clonal dynamics of BRAF^V600E^ melanoma cells’ response to vemurafenib and identify novel targetable vulnerabilities of resistant clones such as oxidative phosphorylation and E2F pathways (141). This approach can be applied to study treatment resistance in the context of BRAF^V600E^ mutated gliomas and will help uncover new mechanisms of tumor cell resistance in the brain. Another cell barcoding strategy was applied to study rare subpopulations, by cloning lentiviral library in which the barcode sequence is incorporated in the 3` untranslated region of the green fluorescence protein (GFP). Through sequencing, barcodes present in resistant samples were used to identify and trace rare, primed cells in the untreated samples. In this way, rare, drug-naive precursors were enriched by FACS and characterized and potential mechanisms of resistance were identified. Interestingly, shortly after drug treatment, these cells highly expressed phosphorylated ERK which confirmed earlier studies that BRAF inhibitor effects on MAPK signaling are only transient (142).

In conclusion, single-cell barcoding can be applied to study treatment resistance in the context of BRAF^V600E^ mutated gliomas and once applied to the brain will help uncover new mechanisms of tumor cell resistance.

## 7 Future perspectives

Model organisms are important substitutes for human studies to facilitate and expedite translation of preclinical studies. Approaches below should be useful for generating next-generation mouse models that better recapitulate the hallmarks and diversity of human cancer (sex, age, race, grade, molecular and clinical features) including gliomas harbored with BRAF^V600E^ mutation.

### 7.1 CRISPR-Cas9 gene editing

CRISPR-Cas9 gene editing technology is now used more often in combination with other systems for developing glioma mouse models (143). Oldrini et al. combined the CRISPR-Cas9 system with the RCAS-TVA system to generate more precisely targeted glioma mouse models for biological and preclinical research including the one for BRAF-mutated glioma by inducing gain-of-function BRAFV637E mutation (orthologous to human BRAF^V600E^) in NSCs (116). However, the CRISPR-Cas9 system suffers from a major limitation of off-target effects, which remain to be addressed.

### 7.2 3D organoid cultures

Recently, 3D organoid culture systems generated from induced pluripotent stem cells (iPSCs), human embryonic stem cells (hESCs), or patient-derived adult stem cells from cancerous tissues have been developed to better capture complex molecular and cellular heterogeneity. In the search for pre-clinical glioma models, remarkable advancements have been made in creating organoid models for gliomas, such as neoplastic cerebral organoids (neoCORs), GLICO, and patient-derived glioblastoma organoids (GBOs) (144–148). These models retain many key features of primary patient tumors, providing accurate models for studying cellular and molecular interactions among and within tumors and thus represent new advanced *ex vivo* models for disease modeling, omics analysis, genetic manipulation, and personalized drug screening. However, the lack of vasculature, stromal and immune cells in these organoids makes it difficult to study tumor-immune microenvironment interactions. Nonetheless, the exciting organoid technology holds tremendous potential to significantly change our understanding of BRAF^V600E^-mutated glioma tumor biology.

### 7.3 Hydrogels and 3D cell culture

Due to low engraftment rates and inconsistent tumor growth of PDX and syngeneic models, various hydrogels have been developed to improve the reliability of mouse models. Although Matrigel is commonly used as a gold-standard hydrogel for 3D cell culture and experimental models (149), batch-to-batch variability in the complex composition of Matrigel can lead to inconsistencies in experimental results, especially when comparing data between different studies using different batches of Matrigel. Therefore, alternative hydrogel scaffolds such as the biomimetic EKGel, tissue-derived extracellular matrix hydrogels, and alginate-based hydrogels have been developed to overcome these limitations (150, 151). Applying one of these advanced hydrogels for culturing BRAF^V600E^-mutated organoids or for encapsulating BRAF^V600E^-mutated tumor cells upon intracranial delivery can improve the consistency of models of BRAF^V600E^ mutated gliomas.

## 8 Conclusions

Preclinical testing of novel therapeutic compounds rarely translates successfully to the clinic due to lack of reproducible cancer models (152). Many promising new anti-cancer treatments are available, especially immunotherapies, that have yet to be assessed preclinically against BRAF^V600E^ mutated CNS malignancies owing to the few preclinical models available. Preclinical results need to be validated in more than one model of a specific tumor type to heighten their predictive capacity. As all the current and next generation *in vitro, ex vivo*, and *in vivo* mouse models have their own individual advantages and disadvantages, selecting the most appropriate model depends on the experimental hypotheses. In order to create models that represent the glioma subtypes found in patients harbored with BRAF^V600E^ mutation, they must be rigorously characterized by genomic, transcriptomic, histopathological, and clinical analyses. Such detailed characterizations are necessary to reveal the level of congruency with the according human disease amongst the distinct models. By using different strategies in combination, clinically relevant mouse models that more closely reflect the complexity and histopathological and genetic diversity of BRAF^V600E^ mutated gliomas can be established. It is important to recognize, however, that markers and features of human tumors are not going to be fully recapitulated in mouse models, due to the obvious differences amongst mice and mice, including in life span, metabolism and physiology, brain microenvironment, and immune responses. Although it has been shown that BRAF^V600E^ alteration is retained in cancer models, molecular data of existing BRAF^V600E^-mutated glioma models are scarce. Thus, it is not known how well these models recapitulate certain pathways activated in human disease, an important question that needs to be addressed is to decide about the utility of each model for answering specific questions.

Although each model has its own distinct strengths and weaknesses, glioma modeling needs to be further refined to accurately mimic the complexity of tumor heterogeneity in human patients and to benefit future clinical translation. Future efforts need to be directed at comparing model organism responses using association analyses, machine learning, pathway enrichment, or meta-analyses, as well as at generating a larger repertoire of preclinical models of BRAF^V600E^-mutated CNS neoplasms that capture the diversity of BRAF^V600E^-mutated CNS neoplasms. The availability and accessibility of next-generation mouse models for BRAF^V600E^-mutated gliomas will have great potential to accelerate our understanding of glioma biology and the development of novel therapeutic strategies.

## Author contributions

YLX wrote the manuscript, created the tables and the figures, and reviewed the final manuscript. DP wrote the manuscript, created the tables, and reviewed the final manuscript. CP conceived the content, wrote the manuscript, created the tables, reviewed and approved the final version of the manuscript. All authors contributed to the article and approved the submitted version.
